# Single cell transcriptome analysis of MCF-7 reveals consistently and inconsistently expressed gene groups each associated with distinct cellular localization and functions

**DOI:** 10.1371/journal.pone.0199471

**Published:** 2018-06-19

**Authors:** Yih-Shien Chiang, Yu-Feng Huang, Mohit K. Midha, Tzu-Han Chen, Hsin-Chieh Shiau, Kuo-Ping Chiu

**Affiliations:** 1 Genomics Research Center, Academia Sinica, Taipei, Taiwan; 2 Institute of Biochemistry and Molecular Biology, National Yang-Ming University, Taipei, Taiwan; 3 College of Life Science, National Taiwan University, Taipei, Taiwan; University of South Alabama Mitchell Cancer Institute, UNITED STATES

## Abstract

Single cell transcriptome (SCT) analysis provides superior resolution to illustrate tumor cell heterogeneity for clinical implications. We characterized four SCTs of MCF-7 using 143 housekeeping genes (HKGs) as control, of which lactate dehydrogenase B (LDHB) expression is silenced. These SCT libraries mapped to 11,423, 11,486, 10,380, and 11,306 RefSeq genes (UCSC), respectively. High consistency in HKG expression levels across all four SCTs, along with transcriptional silencing of *LDHB*, was observed, suggesting a high sensitivity and reproducibility of the SCT analysis. Cross-library comparison on expression levels by scatter plotting revealed a linear correlation and an 83–94% overlap in transcript isoforms and expressed genes were also observed. To gain insight of transcriptional diversity among the SCTs, expressed genes were split into consistently expressed (CE) (expressed in all SCTs) and inconsistently expressed (IE) (expressed in some but not all SCTs) genes for further characterization, along with the 142 expressed HKGs as a reference. Distinct transcriptional strengths were found among these groups, with averages of 1,612.0, 88.0 and 1.2 FPKM for HKGs, CE and IE, respectively. Comparison between CE and IE groups further indicated that expressions of CE genes vary more significantly than that of IE genes. Gene Ontology analysis indicated that proteins encoded by CE genes are mainly involved in fundamental intracellular activities, while proteins encoded by IE genes are mainly for extracellular activities, especially acting as receptors or ion channels. The diversified gene expressions, especially for those encoded by IE genes, may contribute to cancer drug resistance.

## Introduction

Cancer is known to result from progressive accumulation of genomic and epigenomic alterations, leading to dysregulated cell growth [1, 2]. The process of carcinogenesis and subsequent cancer development is strongly enhanced by chromosome instability, a hallmark of cancer, causing the formation of diversified cancer genomes in the cancer mass, each of which drives a cell-specific transcriptome. Through an evolutionary process, cancer cells carrying lethal mutations undergo apoptosis or necrosis, leaving the rest to continue the journey of cancer development in an uncontrolled manner [3, 4]. Due to the advances in Next-Generation Sequencing (NGS) technologies, cancer genomes and associated transcriptomes can be analyzed at single cell level [5, 6]. Due to the fact that it is able to provide a superior resolution as enhanced by NGS, single cell transcriptome (SCT) analysis has a strong potential to facilitate our understanding of cancer evolution and its transcriptional heterogeneity [7, 8], which is believed to be associated with acquired drug resistance [9]. However, although with a great potential [10], SCT data from cancerous cells remain very limited.

Here, we conducted SCT sequencing on MCF-7 breast cancer cell line to reveal their transcriptional diversity and potential clinical implications. MCF-7 is one of the most widely used metastatic breast cancer cell line. It retains several characteristics of differentiated mammary epithelium (https://www.atcc.org/Products/All/HTB-22.aspx#characteristics). On the other hand, breast cancer cells such as those characterized as HER2-positive and TNBC (Triple Negative Breast Cancer) have already experienced intensive selection process to abolish the expressions of some key genes, which may further affect the expressions of many other genes to make the transcriptome more subtype-specific. We prefer to use MCF-7 due to the fact that it represents a more general form of breast cancer and has a better potential to express both consistently expressed and inconsistently expressed genes. The analysis employs RNA-Seq approach [11], in conjunction with Tophat and Cufflinks software encompassed in UCSC Galaxy pipeline. To evaluate the reliability of our approach, we used 143 housekeeping genes (HKGs) agreed by two independent groups [12, 13], as control. HKGs, originally defined as genes with constitutive transcriptional activation in all cells [14], have frequently been used as reference to estimate gene expression levels [13, 15]. Moreover, *LDHB* gene, encoding for lactate dehydrogenase B, is silenced in MCF-7 by promoter hypermethylation and thus can serve as another layer of control [16, 17].

We split the expressed genes into consistently expressed (CE) (i.e., expressed in all SCTs) and inconsistently expressed (IE) (i.e., expressed only in some but not all SCTs) genes and conducted serial analyses on these two groups together with HKGs. Results indicated a bipartite transcriptional pattern in MCF-7 single cells, with consistently expressed genes mainly coding for proteins involved in intracellular activities and inconsistently expressed genes coding for extracellular proteins, including receptors and ion channels. We suspect that diversified expression of IE genes may act as a frontline protection of cancer cell from the attack of cancer drugs, due to the fact that it is essential for cancer drugs to interact with extracellular and/or membrane-bound proteins and the absence in expression in some cells would render the cancer as a whole to survive cancer drug treatment.

## Materials and methods

### Cell culture and single cell isolation

MCF-7 (BCRC 60436, Lot-01337) was purchased from Bioresource Collection and Research Center of Taiwan and cultured in DMEM medium (GIBCO 11955–065) supplemented with 10% (v/v) fetal bovine serum (GIBCO 26140–079) and 5 mg/ml insulin (Sigma I0516), in the presence of penicillin (100 unit/ml) and streptomycin (100 μg/ml). Cell culture was maintained inside a 37°C humidified incubator containing 5% CO_2_. Culture media were changed every 3 days. Single cells were picked up by mouth pipetting under a microscope and transferred into 0.2 ml thin-walled PCR tubes, each containing 4.0 μl of cell lysis buffer. Volume of solution carryover was kept below 0.5 μl to minimize its impact on experimentation.

### Sequencing library construction and sequencing

Sequencing libraries were constructed using ABI protocol [7], but with some modifications: 1) SuperScript II, instead of SuperScript III, was used as the reverse transcriptase during reverse transcription with UP1 primer; 2) incubation conditions were changed from “50^°^C for 30 min” to “42^°^C for 50 min”; and 3) duration time of the first PCR cycle was extended from 6 min to 8 min. PCR products were displayed by 1.5% agarose gel electrophoresis and 0.5–3 kb cDNA molecules were isolated for sequencing library construction. All SCT libraries were sequenced with SOLiD 3 for 35 ligation cycles (eq. 35 bp in length).

### RNA-Seq data processing and analysis

Galaxy pipeline was used for SCT data analysis. Data processing comprised the following steps: quality sequence extraction, decontamination, mapping with Tophat, and gene annotation with Cufflinks [18]. In more detail, we used a cutoff value of 5 in the ‘remove reads containing color quality below this value’ parameter selection box to select quality reads. Sequence reads containing vector sequences or homopolymers (≥ 9 bp) were discarded. Quality reads were mapped to hg19 with Tophat originally designed for short read alignment for RNA-Seq experiments. We allowed two mismatches for each 35 bp read. ‘Minimum isoform fraction: filter out junctions supported by too few alignments’ was set to zero, and all other parameters were retained as default. Sequence reads with ≤ 10 hits were used and subjected to Cufflinks for annotation. To maximize sensitivity, both ‘min-isoform fraction’ and ‘pre-MRNA fraction’ were set to 0.0, and the parameter for ‘max-intron-length’ was changed from 300,000 to 500,000. To exclude singleton transfrags (transcribed fragments), we changed ‘min-frags-per-transfrag’ from 10 to 2 in program coding. All the other Cufflinks parameters were retained as default.

### Cross-library comparison and gene level analysis

The expression levels of all expressed genes were compared between libraries by scatter plotting and the degree of overlap in transcript isoforms, gene IDs, and gene symbols were analyzed based on results generated by Cufflinks. For overlap analysis, we checked the presence of the same transcript isoform, gene ID, and gene symbol for all two-library combinations, and calculated the percentage of common objects over the total number of objects for each library in every combination. Expressed genes, including those in reference HKGs, were divided into CE and IE groups.

### Gene ontology analysis

We employed Gene Ontology to analyze CE and IE genes. Biological Process (BP), Cellular Component (CC) and Molecular Function (MF) are the three fundamental domains of Gene Ontology. The controlled vocabularies were built as a directed acyclic graph (DAG), with the top hierarchy of GO tree defined more general terms and the more specific terms were obtained when traversed down the tree.

Before the analysis, both gene_ontology.1_2.obo file (http://www.geneontology.org/ontology/obo_format_1_2/gene_ontology.1_2.obo) and gene2go.gz (ftp://ftp.ncbi.nlm.nih.gov/gene/DATA/gene2go.gz) were downloaded from Gene Ontology website and NCBI FTP site, respectively. The gene_ontology.1_2.obo file was parsed into three individual domain files and gene2go file was served as reference for Gene Symbol-GO ID correlation. All RefSeq genes (13,764 total) were associated with corresponding GO terms in various domains based on the gene_ontology.1_2.obo and gene2go.

To perform statistical test, the number of genes in an annotation file (i.e., BP, CC and MF), the number of genes annotated to GO term, a list of genes from user input and genes annotated to each GO term are needed. For each GO term, we performed hypergeometric test to obtain significant GO term based on p-value. Then, we ranked each GO term based on its smallest p-value. For each selected group (CE or IE), the hypergeometric test was performed to obtain the top GO terms from three different domains, and the GO terms from CE and IE groups were compared with each other.

### Pathway analysis

We chose BioCarta, which provides clear overview of pathways in suitable scale, for pathway analysis. It associates genes with pathways and we used those genes as our sampling for further calculation. We annotated CE genes to pathways and calculated the gene coverage rate for each pathway, shown as “percent” in table. Based on hypergeometric distribution, which is a discrete probability distribution, we get the “p-value” from formula

f(k;N,m,n)=∑k+1m(mk)(N−mn−k)(Nn),

where N equals total number of Refseq genes in BioCarta, n equals total number of CE genes in BioCarta, m equals the number of genes in each pathway, and k equals the number of CE genes matched to the pathway. To identify the most significantly annotated pathways, we set percentage > 80% and p-value < 10^−3^.

## Results and discussion

### Library statistics

A total of five single cells (sC4, sC5, sC6, sC7 and sC8) were initially isolated from culture for experimentation. Among those, sC7 was found to have much lower P2#/P1# ratio (48%) compared to the rest (78–86%) and was excluded, so to minimize bias [19].

As described in SOLiD 3 Instrument Operation Guide (page 174) provided by Applied Biosystems (http://tools.thermofisher.com/content/sfs/manuals/4407430b.pdf), P2#/P1# ratio is defined as “the frequency of template-positive beads (P2#) relative to total beads (P1#) deposited on the slide; this metric is also referred to as “% P2 Positive” value”.

In more detail, SOLiD systems use two different adaptors, P1 and P2, to ligate to the ends of target DNA fragments, which are subsequently denatured into single-stranded templates for sequencing library construction. To make fluorescent signal detectable, clusters are built from adaptor-ligated single-stranded templates on magnetic beads using a procedure called “emulsion PCR” which contains millions of PCR reactions (each contains all PCR reagents, with P2 adaptor in excess and with none or limited number of beads, as following Poisson distribution) taking place in micro-scale aqueous droplets separated by mineral oil. In each PCR reaction, the P1 region of single-stranded template is annealed to the oligos pre-anchored on bead, while the P2 region is managed to reside on the other end. After emulsion PCR, beads with clusters are enriched, while beads without clusters are discarded. Since the presence of P1 is essential for making clusters on beads, P1# is used to represent total beads. On the other hand, although enriched, some beads may not have P2 on the other end (e.g., it may have P1 instead). As such P2#/P1# ratio is a metric to indicate the quality of template construction, where P2# represents the desired template-positive beads. High and balanced P2#/P1# ratios are desired for reliable analysis.

Prior to analysis, a series of examinations were conducted to estimate the degree of balance between SCT libraries. The sequencer generated 45.7–53.4 million 35 bp reads per library (Table 1). Over half of these reads passed quality checking, accounting for 8.1–8.8 million unique reads (uReads) per library. Since PCR increases the copy number of uRead, we calculated “total count-to-uRead” (T2U) ratio and used it as an indicator to estimate the degree of PCR-induced copy number increase. The T2U ratios fell within a narrow range between 3.0–3.3, indicating a balanced PCR amplification among these libraries. Both numbers of mapped reads and numbers of mapped uReads also fell within a narrow range. Reads with 1–10 mapped locations were then used for further analysis. They were annotated to 10,380–11,487 RefSeq genes after the exclusion of those mapped by singletons. These results indicate that preparations of these SCT libraries were well-balanced.

**Table 1:** Library statistics.

Description	sC4	sC5	sC6	sC8
#Original raw reads	47,563,622	48,815,843	53,363,798	45,762,729
#Quality reads	25,981,470 (54.6%)	27,144,452 (55.6%)	26,920,303 (50.4%)	26,926,581 (58.8%)
#Unique reads (uReads)	8,758,507	8,484,167	8,112,309	8,362,464
T2U ratio	3.0	3.2	3.3	3.2
#Mapped reads (1≤ #Mapped locations ≤ 10)	17,940,246 (69.1%)	20,349,372 (75.0%)	18,768,185 (69.7%)	18,815,190 (69.9%)
#Mapped uReads (1≤ #Mapped locations ≤ 10)	4,379,627 (50.0%)	5,249,695 (61.8%)	4,555,764 (56.2%)	4,482,719 (53.6%)
#Genes (singleton included)	12,178	12,721	11,745	13,522
#Genes (singleton excluded)	11,423	11,487	10,380	11,306
#Genes mapped by singletons	755	1,234	1,365	2,216
#Novel clusters	47,544	70,099	52,711	110,671

### Expressions of the 143 reference housekeeping genes

Expressions of the 143 reference HKGs served as a reference to evaluate the reliability of the SCT analysis (S1 Table). Although HKGs may be redefined in cancer cells, their expressions, in general, are expected to be maintained within a fair range in single cells, due to the fact that their expressions are essential for maintaining cellular structure and functions.

Among the 143 HKGs, 140 were found consistently expressed (and thus included in the CE group), while *LDHB* was not expressed for all libraries as expected and *MSN* and *PIM1* were expressed at barely detectable levels in some but not all single cells (and thus included in the IE group) (Table 2). *MSN* encodes moesin protein that mediates the association of cell membrane and actin, and the interaction with extracellular matrix, while *PIM1* is a proto-oncogene encoding a serine/threonine kinase.

**Table 2:** Expression of the 143 reference housekeeping genes.

Libraries \ HKGs	# expressed HKGs (143 total)	Unexpressed HKGs
sC4	141	*LDHB*, *MSN*
sC5	142	*LDHB*
sC6	140	*LDHB*, *MSN*, *PIM1*
sC8	142	*LDHB*

**Fig 1:**
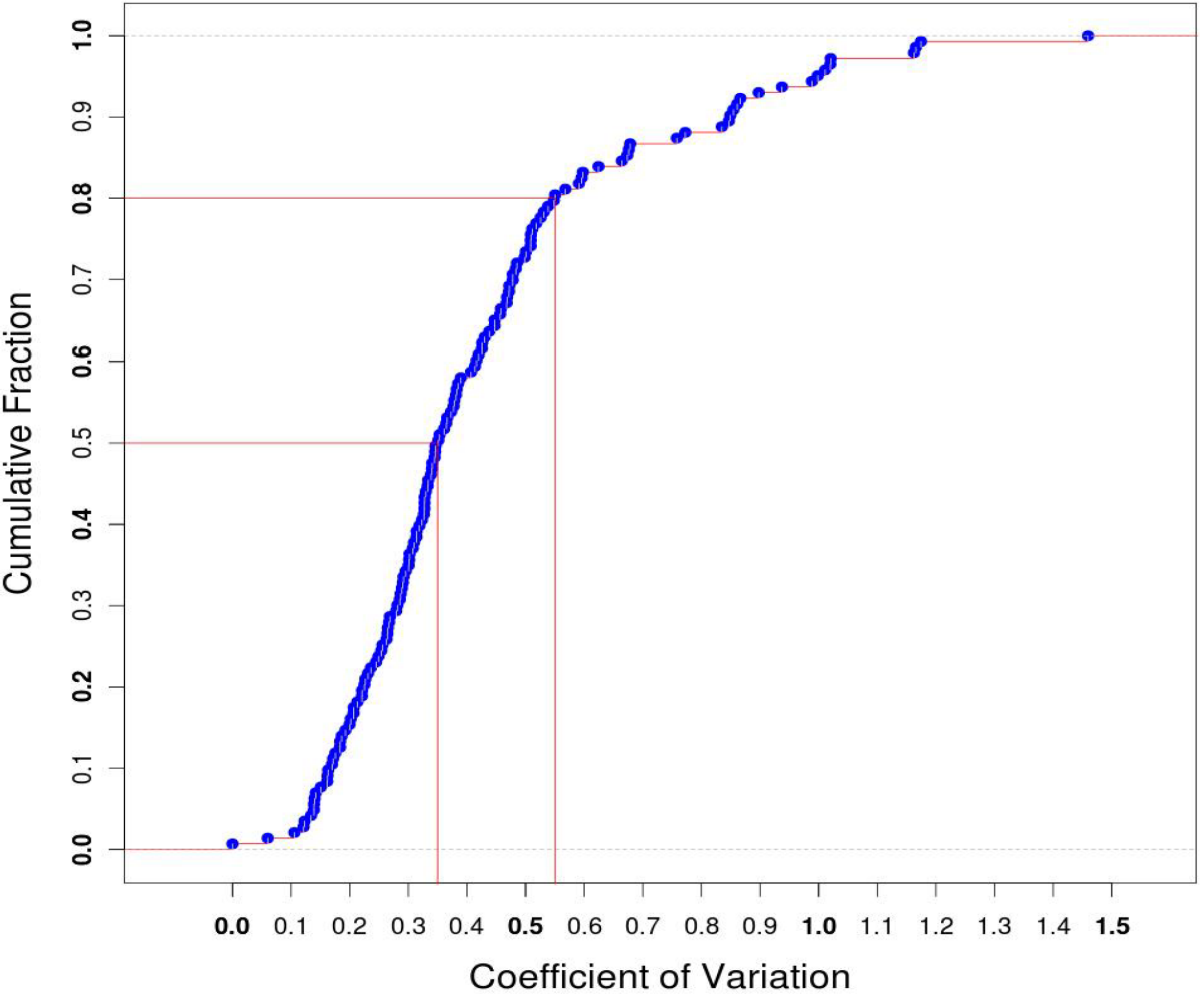
Distribution of coefficient of variation (CV) of the 143 reference HKGs. The coefficient of variation was calculated for the expressions of each 143 reference HKGs in four SCT libraries and then plotted against their corresponding percentage. As shown in the figure, about 80% of the CVs were less than 0.55.

The expression levels of each reference HKG in four SCT libraries were compared and used to calculate the coefficient of variation (CV) which represents the degree of fluctuation in expression. About 80% of the reference HKGs had CV values less than 0.55 (Fig 1), indicating a stable expression of these HKGs among single cells.

Noticeably, the expressions of the *GAPDH* gene, which encodes glyceraldehyde-3-phosphate dehydrogenase and which is one of the HKGs most frequently used for the quantification of mRNA and protein expressions, fell within a narrow range between 5,078.3–6,658.2 FPKM, suggesting a strong reliability of the SCT approach.

### Transcriptome similarities among SCT libraries

**Fig 2:**
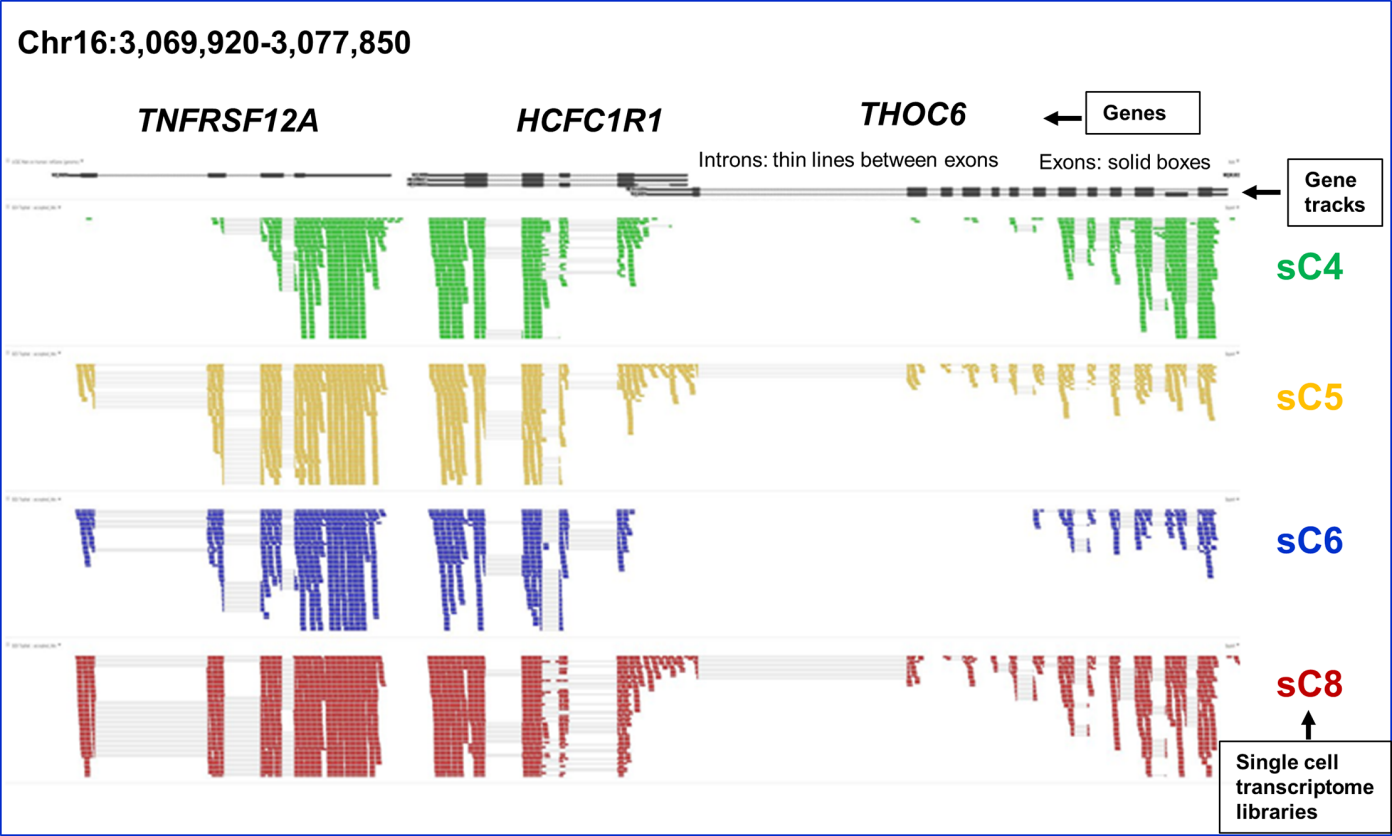
An example of single cell transcriptome profiles produced by Galaxy. Genome-wide RNA-Seq shotgun sequences of sC4, sC5, sC6 and sC8 single cell transcriptomes (highlighted in different colors) were analyzed by Galaxy pipeline which adopts Tophat for mapping (against hg19) and Cufflinks for transcript isoform identification. The height of each cluster represents its relative expression level. Some peaks are not shown completely due to space constraint. Introns (thin grey lines) between exons and intergenic regions (thick grey lines) are barely seen between exons.

Due to the fact that these single cells were all derived from the same cell line, we expected a high degree of similarity among their SCTs. The similarities among these SCTs were first exemplified graphically prior to the characterization of transcriptional heterogeneity (Fig 2). The similarity is not only shown in the state of ON or OFF of transcription, but also in expression level (shown as heights in the figure).

**Fig 3:**
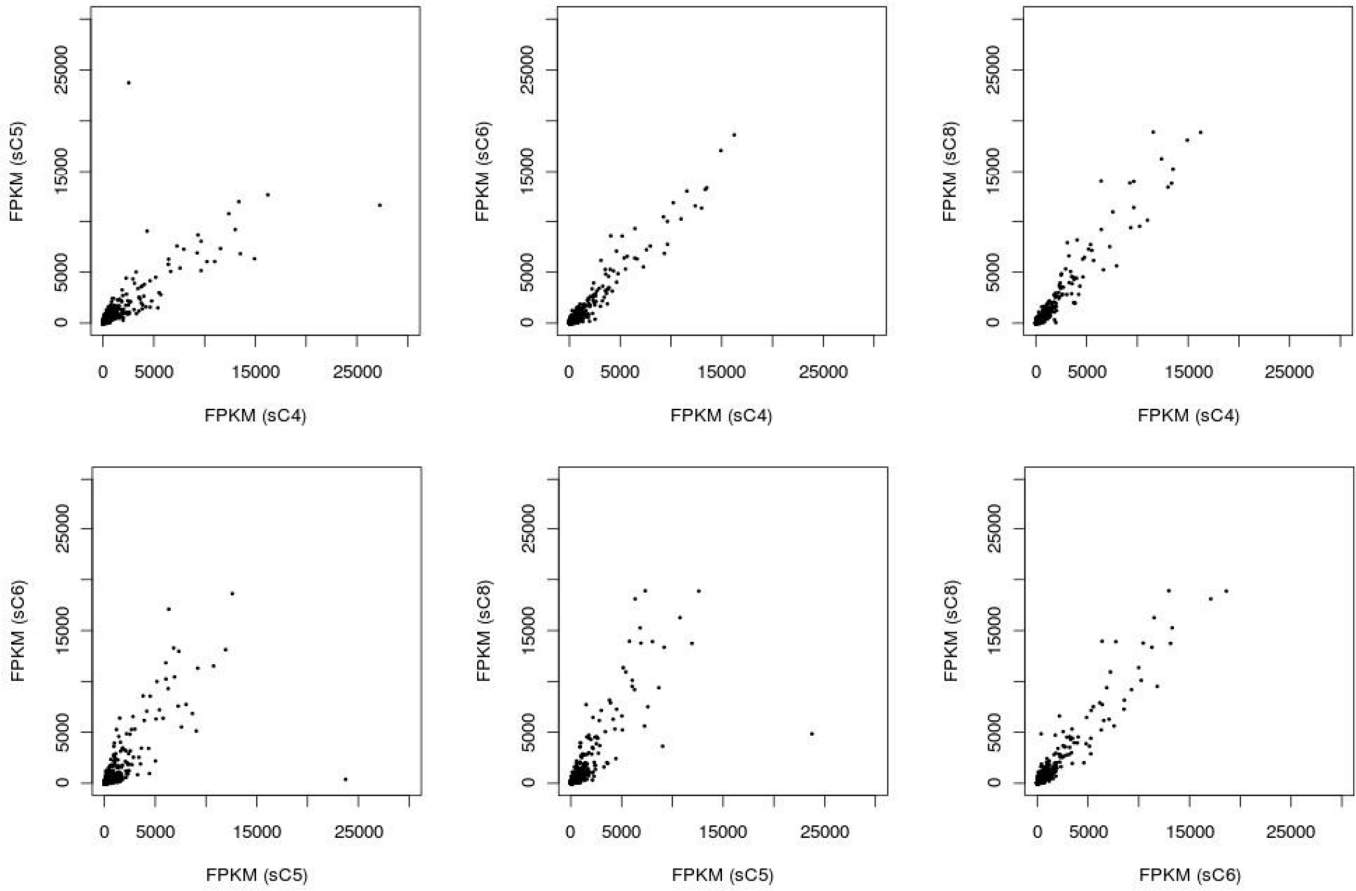
Scatter plots to show the correlation in gene expression levels for all two-library combinations of all four SCT libraries. The gene expression levels, in FPKM, of all expressed genes are plotted for any two-library combination of the SCT libraries.

We then employed scatter plotting to analyze the correlation in gene expression levels between SCTs (see S1 Table). Result indicates a linear correlation in gene expression between any two-library combination of the SCT libraries (Fig 3).

**Fig 4:**
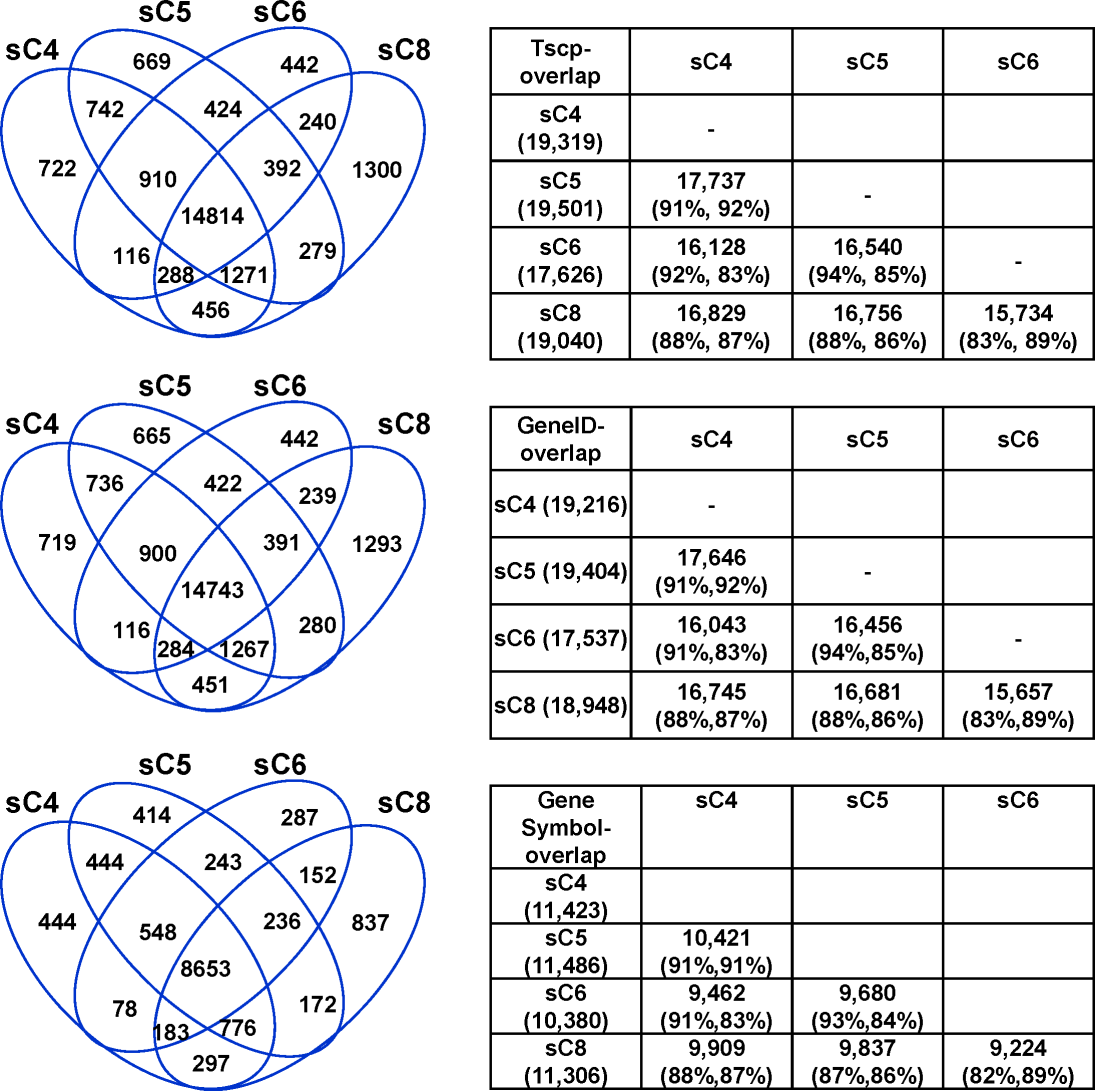
Overlaps in gene transcript isoforms, gene IDs and gene symbols among the SCTs. The numbers (left) and percentages (right) of overlaps in transcript isoforms (top), gene IDs (middle), and gene symbols (bottom) among the SCT libraries are displayed with Venn Diagrams and tables. Tables on the right show the numbers of overlap in two-library combinations together with percentages for the library on the left (left within the parentheses) and the library on top (right within the parentheses).

To gain more insight of the similarity among these SCTs, we then analyzed the overlap in transcript isoforms among the SCTs. For the analysis, gene IDs and transcript isoforms were identified by Cufflinks. Gene IDs were then assigned to their corresponding gene symbols using UCSC database. Results indicated a high degree of similarity in transcript isoforms, ranging between 83–94, 83–94 and 82–93 for the overlaps in transcripts, gene IDs and gene symbols, respectively (Fig 4), further indicating a high degree of reliability of the experimental and analytical procedures.

Thus, both linear scatter plots and high percentage of overlaps in transcripts indicate a high degree of similarity among the SCTs.

### The consistently expressed genes, inconsistently expressed genes and the 142 expressed reference housekeeping genes are expressed at distinct levels

**Fig 5:**
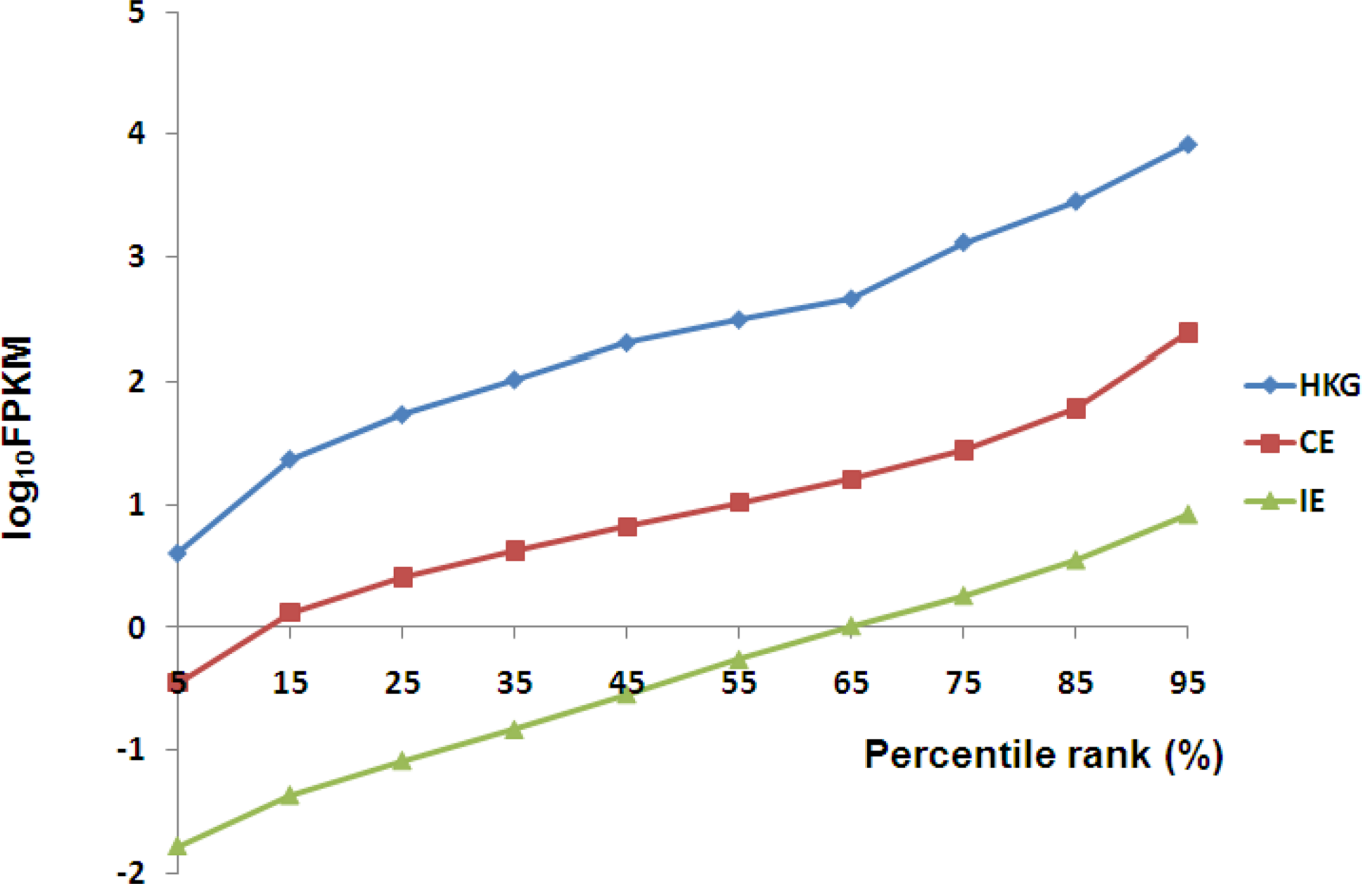
Expression level distributions for the 142 expressed reference HKGs, CE group, and IE group. For every gene in every group, the expression levels in all SCTs were averaged and sorted. FPKM values at 5%, 15%, 25%, 35%, 45%, 55%, 65%, 75%, 85% and 95% were sampled and converted by log_10_ and plotted for cross-library comparison.

To analyze transcriptional diversity among the SCTs, expressed genes were first split into consistently expressed (8,653 total, including 140 consistently expressed HKGs), inconsistently expressed genes (5,111 total, including 2 inconsistently expressed HKGs) and the 142 expressed HKGs as a whole. The expression levels of each gene in each group were averaged and sorted ascendingly. FPKM values at 5%, 15%, 25%, 35%, 45%, 55%, 65%, 75%, 85% and 95% were sampled and converted by log_10_ and plotted side by side for comparison. Results demonstrate distinct expression levels (Fig 5), but with similar distribution patterns. Among these groups, 142 HKGs are expressed at the highest level, followed by CE, and then by IE. The mean values of expression levels of the 142 expressed HKGs, CE and IE are 1,612.0, 88.0 and 1.2, respectively, while the overall expression levels averaged at 54.6 FPKM. Thus, the mean value of CE gene expression levels is approximately 73 fold higher than that of the IE group.

### The expression levels of consistently expressed genes vary much more significantly compared to that of inconsistently expressed genes and housekeeping genes

**Fig 6:**
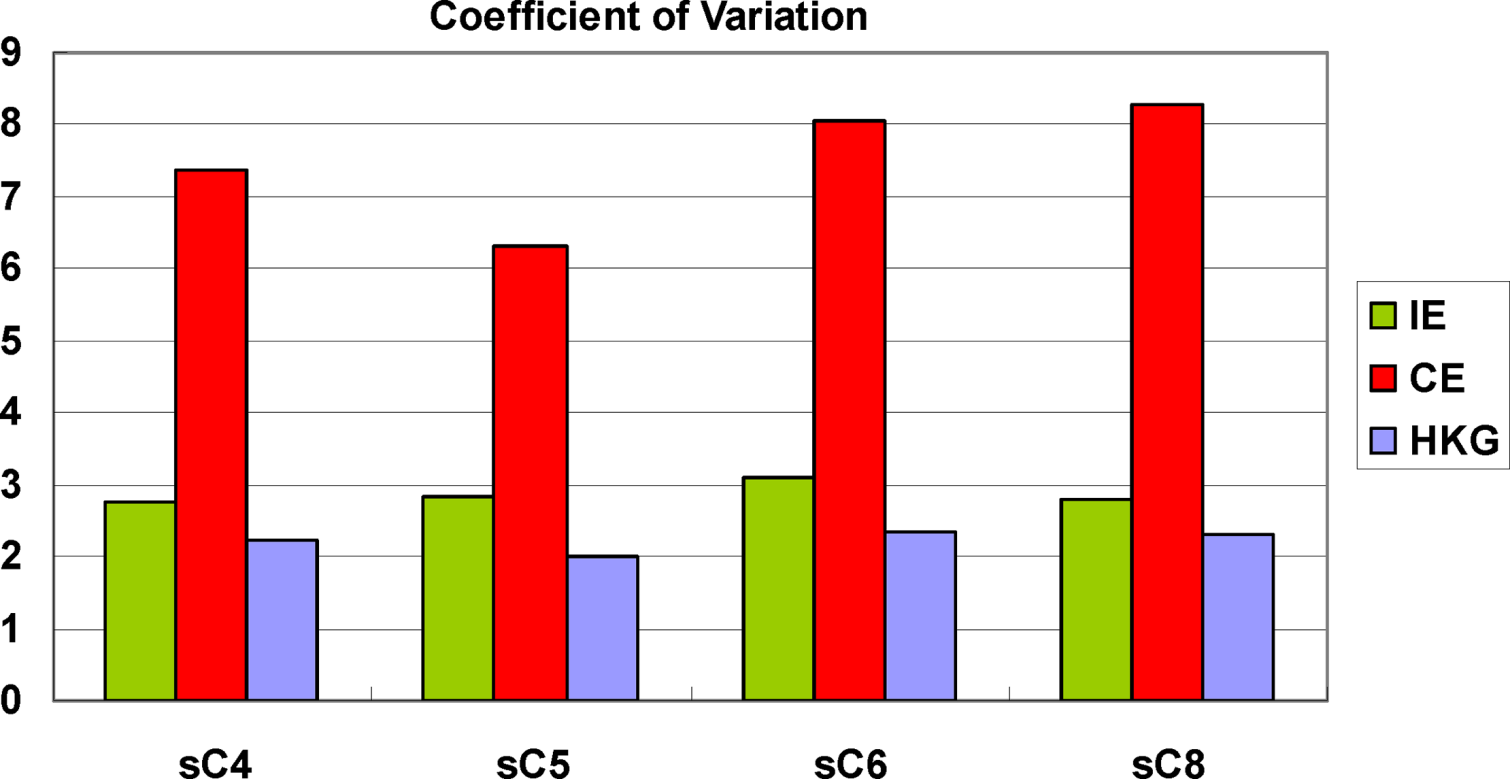
Comparison of coefficient of variation for HKGs, CE and IE genes. CVs were calculated for all three gene groups, including housekeeping genes, consistently expressed genes, and inconsistently expressed genes and displayed separately for all single-cell library.

We then analyzed the variation in gene expression levels for every group in every single cell library. Interestingly, CV of the CE group was significantly higher than that of IE group and HKGs across all SCT libraries (Fig 6). We do not know the biological meaning of this phenomenon.

### Gene ontology analysis revealed distinguishable functional categories for consistently expressed genes and inconsistently expressed genes

**Fig 7:**
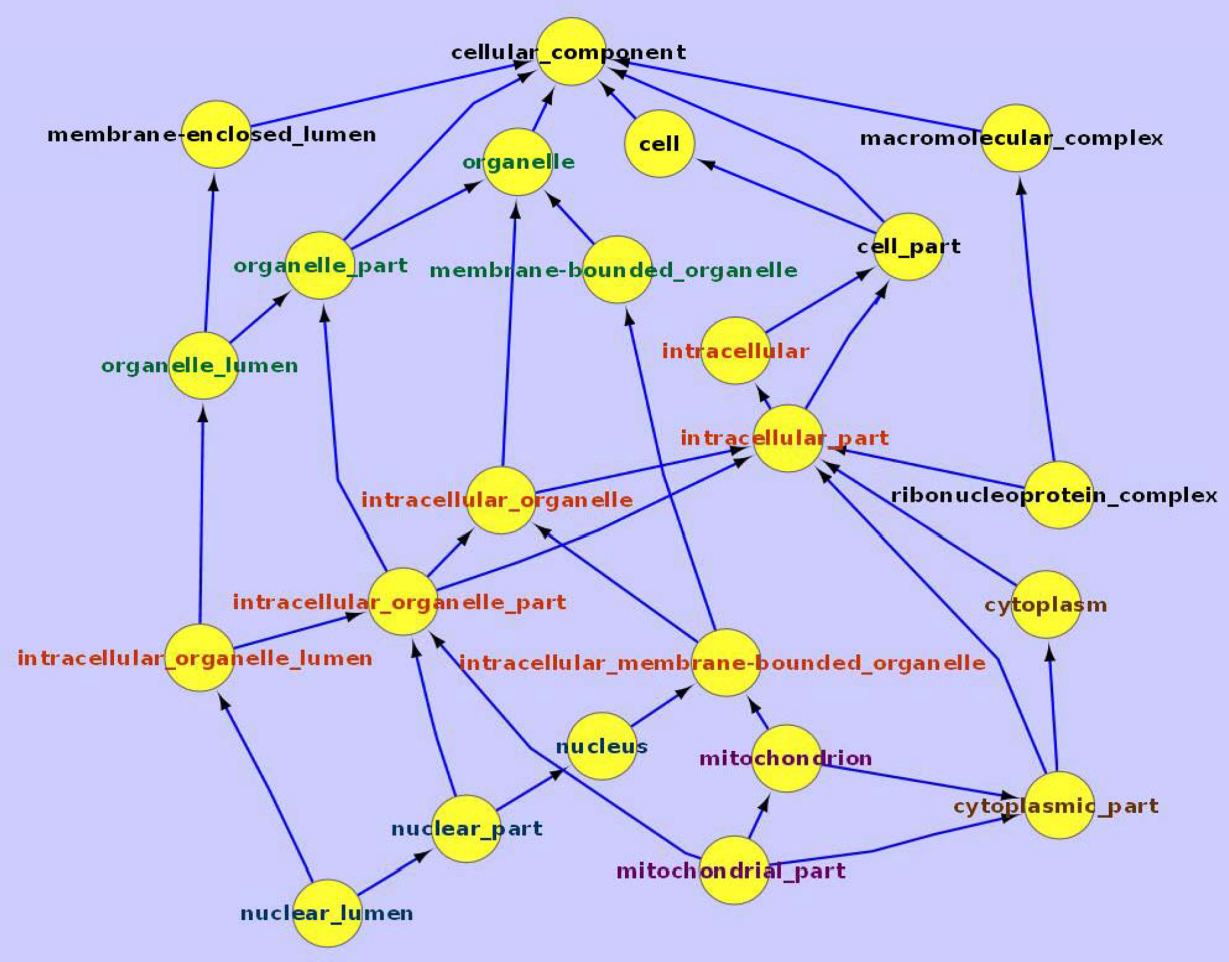
GO cellular component analysis for the consistently expressed (CE) genes. Genes consistently expressed in all four single cell transcriptome libraries are analyzed using Gene Ontology cellular component database. Components associated with major terms are highlighted by color, e.g., ‘organelle’ in green, ‘intracellular’ in red, and ‘mitochondrion’-related in purple.

We conducted GO analysis to gain insight of the biological functions of CE and IE genes. Results indicate that CE genes are more likely to encode proteins associated with intracellular structures such as membrane-bound organelles, organelle- and nuclear-associated structures, cytosol, cytoplasmic components, etc. (Fig 7). Their associated biological processes include translation, RNA (esp. mRNA) processing and metabolic activities, and their associated molecular functions are mainly related to protein and RNA binding and structural constituents of ribosome.

**Fig 8:**
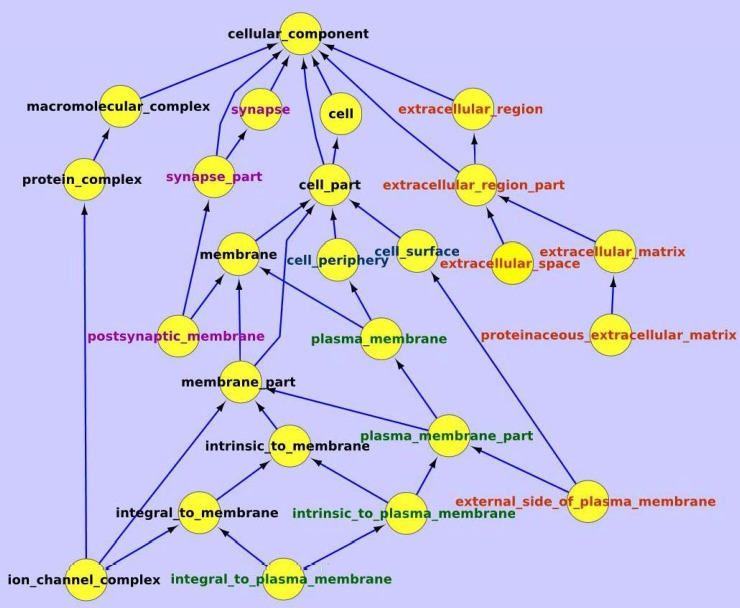
GO cellular component analysis for the inconsistently expressed (IE) genes. Genes inconsistently expressed in the four single cell transcriptome libraries are analyzed using Gene Ontology cellular component database. Components associated with major terms are highlighted with color, e.g., ‘synapse’ in purple, ‘plasma membrane’ in green, and ‘extracellular or external’ in red.

On the other hand, IE genes encoded proteins are more likely to be involved in extracellular structures such as cell periphery, plasma membrane, extracellular space and matrix, ion channel, etc. (Fig 8). Their associated biological processes include system process, multicellular organismal process, cell-cell signaling, etc., and their associated molecular functions are mainly related to receptor and channel protein activities (also see S2 and S3 Tables).

Thus, there is an evident bipartite feature in gene expression within every single cell and this bipartite feature is not limited to either healthy or diseased cells. However, since proteins encoded by IE genes are more involved in extracellular activities, inconsistent expression among single cells is expected to play a role in drug resistance in cancer.

### Pathway analysis for the CE genes

Global pathway analysis using BioCarta pathway database indicated that many of the CE genes are participated in multiple key signaling pathways (including mTOR, MAPK, proteasome, EIF2, KREB, HIF, and RAS), together with glycolysis pathway, AKT pathway, and a number of growth factor/RTK pathways (including PDGF, EGF, IGF, VEGF, and insulin) (S3 Table). Many of these pathways (to name a few, HIF, VEGF, AKT, and RAS) are known to be tightly associated with cancer metabolism [20, 21]. We did not perform pathway analysis for IE genes because they are mainly extracellular structural proteins.

## Discussion

Tumor cell heterogeneity is one of the key factors responsible for drug resistance, which in turn plays a key role for cancer therapy failure [9, 22]. Previous cancer investigations mainly used clinical tissues composed of mixed cancer cell populations, and thus the molecular interactions and interchange of proteins between individual cancer cells could not be well understood. Single cell sequencing would enhance our understanding of tumorigenesis and cancer treatment. Our study provides further information to explain part of the mechanisms associated with cancer drug resistance.

In an attempt to identify the transcriptional heterogeneity of protein-coding genes, we analyzed MCF-7 transcriptomes at single cell level. We split the expressed genes into consistently expressed genes and inconsistently expressed genes and studied the attributes of each group, followed by gene level and pathway level analyses. We show that HKGs, CE genes, and IE genes are expressed at distinguishable levels. Furthermore, in general, IE gene expressions showed less fluctuation than that of CE genes and this phenomenon was observed across all SCT libraries. We do not know the in-depth biological meaning underlining this phenomenon. However, we cannot exclude the possibility that fluctuation in transcriptional level of the CE genes may also contribute to the variation in drug resistance.

Interestingly, GO analysis indicates that proteins encoded by CE genes are more associated with intracellular physiological functions, while proteins encoded by IE genes are mainly involved in extracellular structures. Since structural heterogeneity, together with associated functional heterogeneity, is a well-known phenomenon associated with cancer drug resistance, diversified expression of extracellular proteins in MCF-7 single cells should result in differential drug responses and thus contribute to cancer drug resistance. It is understandable that intracellular proteins are more related to the core physiological functions of a cell and thus need to be expressed constitutively. On the other hand, extracellular proteins are more involved in cellular integrity. Since there are many types of proteins in the cellular membrane and thus some can be omitted or facultatively expressed in some cells without causing lethal consequence. Cancer cells seem to take advantage of this feature to minimize the impact of drug treatment.

## Supporting information

S1 Table This Excel file lists all expressed genes (including CE, IE and 143 HKGs), their expression levels (in FPKM) in all four single cell transcriptome libraries, their averages, standard deviations and coefficient of variations (CVs). (XLSX)

S2 Table This file shows the results of GO analysis for CE and IE groups. (PDF)

S3 Table This file shows the results of BioCarta pathway analysis of CE genes. (Pathway analysis for IE is skipped because they are mainly extracellular proteins).
(PDF)
