# Supplementary material for: Single cell transcriptome analysis of MCF-7 reveals consistently and inconsistently expressed gene groups each associated with distinct cellular localization and functions
Source: PLoS One. 2018 Jun 19;13(6):e0199471. doi: 10.1371/journal.pone.0199471 (PMC6007910; doi:10.1371/journal.pone.0199471)
Supplement: S2 Table — (PDF) [file pone.0199471.s002.pdf]

Supplementary Table S2:
GO analysis for CE and IE genes

CE.1) Consistently Expressed Genes: Cellular Component

GOID	GO #gene	CE #gene	p-value	GOterm
GO:0044424	8679	6073	1.24E-92	intracellular part
GO:0005622	8875	6181	7.25E-90	intracellular
GO:0043227	6814	4900	6.92E-81	membrane-bounded organelle
GO:0043231	6809	4895	2.91E-80	intracellular membrane-bounded organelle
GO:0043226	7463	5288	1.73E-74	organelle
GO:0043229	7456	5283	2.85E-74	intracellular organelle
GO:0044446	4610	3443	2.84E-73	intracellular organelle part
GO:0044422	4648	3464	7.43E-72	organelle part
GO:0005737	6372	4560	2.16E-62	cytoplasm
GO:0070013	2246	1767	5.98E-55	intracellular organelle lumen
GO:0031974	2299	1802	4.27E-54	membrane-enclosed lumen
GO:0043233	2269	1777	9.95E-53	organelle lumen
GO:0044444	4619	3380	1.55E-52	cytoplasmic part
GO:0044428	2166	1693	9.51E-49	nuclear part
GO:0030529	489	451	7.87E-47	ribonucleoprotein complex
GO:0005739	1148	947	5.53E-43	mitochondrion
GO:0031981	1915	1491	2.44E-40	nuclear lumen
GO:0032991	2695	2019	3.56E-36	macromolecular complex
GO:0044429	598	516	3.24E-33	mitochondrial part
GO:0005634	4313	3090	1.78E-31	nucleus
GO:0005730	981	784	1.78E-26	nucleolus
GO:0031967	616	516	2.43E-26	organelle envelope
GO:0005840	193	184	1.84E-24	ribosome
GO:0031975	623	517	1.94E-24	envelope
GO:0005829	1644	1243	2.27E-23	cytosol
GO:0005654	1127	879	2.84E-23	nucleoplasm
GO:0043228	2483	1816	2.21E-22	non-membrane-bounded organelle
GO:0043232	2483	1816	2.21E-22	intracellular non-membrane-bounded organelle
GO:0005740	407	349	1.25E-21	mitochondrial envelope
GO:0005623	10509	6938	1.65E-20	cell
GO:0044464	10509	6938	1.65E-20	cell part
GO:0031966	389	333	2.01E-20	mitochondrial membrane
GO:0031090	1630	1212	2.98E-18	organelle membrane
GO:0005743	276	242	5.49E-18	mitochondrial inner membrane
GO:0019866	301	260	2.75E-17	organelle inner membrane
GO:0005759	241	212	2.87E-16	mitochondrial matrix
GO:0044445	137	128	3.26E-15	cytosolic part
GO:0044451	618	488	7.81E-15	nucleoplasm part
GO:0016604	208	183	3.15E-14	nuclear body
GO:0005681	135	125	4.75E-14	spliceosomal complex
GO:0043234	2190	1566	7.26E-13	protein complex
GO:0016607	114	106	2.02E-12	nuclear speck
GO:0044455	118	109	3.19E-12	mitochondrial membrane part
GO:0022626	75	73	7.89E-12	cytosolic ribosome
GO:0015934	63	62	5.80E-11	large ribosomal subunit
GO:0070469	63	62	5.80E-11	respiratory chain
GO:0005746	58	57	4.64E-10	mitochondrial respiratory chain
GO:0015935	57	56	7.02E-10	small ribosomal subunit
GO:0000502	59	57	4.96E-09	proteasome complex
GO:0010008	205	170	9.11E-09	endosome membrane

GO:0044440	205	170	9.11E-09	endosomal part
GO:0005747	39	39	5.21E-08	mitochondrial respiratory chain complex I
GO:0030964	39	39	5.21E-08	NADH dehydrogenase complex
GO:0045271	39	39	5.21E-08	respiratory chain complex I
GO:0022625	37	37	1.23E-07	cytosolic large ribosomal subunit
GO:0005768	335	261	1.64E-07	endosome
GO:0000313	49	47	2.58E-07	organellar ribosome
GO:0005761	49	47	2.58E-07	mitochondrial ribosome
GO:0005635	216	174	3.67E-07	nuclear envelope
GO:0035097	58	54	5.94E-07	histone methyltransferase complex
GO:0005694	466	349	2.25E-06	chromosome
GO:0034708	59	54	2.55E-06	methyltransferase complex
GO:0042470	82	72	2.71E-06	melanosome
GO:0048770	82	72	2.71E-06	pigment granule
GO:0008287	41	39	5.74E-06	protein serine/threonine phosphatase complex
GO:0022627	35	34	5.79E-06	cytosolic small ribosomal subunit
GO:0000785	194	153	1.81E-05	chromatin
GO:0005643	67	59	1.82E-05	nuclear pore
GO:0044427	390	291	2.59E-05	chromosomal part
GO:0015030	42	39	2.96E-05	Cajal body
GO:0046930	76	65	5.59E-05	pore complex
GO:0031965	128	103	9.66E-05	nuclear membrane
GO:0012505	1179	823	1.72E-04	endomembrane system
GO:0045259	20	20	1.86E-04	proton-transporting ATP synthase complex
GO:0000123	66	56	2.90E-04	histone acetyltransferase complex
GO:0000790	70	59	2.92E-04	nuclear chromatin
GO:0016585	89	73	3.30E-04	chromatin remodeling complex
GO:0005793	48	42	4.28E-04	ER-Golgi intermediate compartment
GO:0000314	18	18	4.39E-04	organellar small ribosomal subunit
GO:0005753	18	18	4.39E-04	mitochondrial proton-transporting ATP synthase complex
GO:0005763	18	18	4.39E-04	mitochondrial small ribosomal subunit
GO:0030530	18	18	4.39E-04	heterogeneous nuclear ribonucleoprotein complex
GO:0033116	18	18	4.39E-04	ER-Golgi intermediate compartment membrane
GO:0009295	34	31	5.00E-04	nucleoid
GO:0005741	91	74	5.03E-04	mitochondrial outer membrane
GO:0031901	43	38	5.22E-04	early endosome membrane
GO:0042645	33	30	7.06E-04	mitochondrial nucleoid
GO:0000228	183	139	9.50E-04	nuclear chromosome
GO:0005769	117	92	9.96E-04	early endosome
GO:0005625	226	169	1.04E-03	soluble fraction
GO:0031968	106	84	1.05E-03	organelle outer membrane
GO:0016469	40	35	1.33E-03	proton-transporting two-sector ATPase complex
GO:0048471	276	203	1.48E-03	perinuclear region of cytoplasm
GO:0030684	15	15	1.59E-03	preribosome
GO:0044454	149	114	1.69E-03	nuclear chromosome part
GO:0030532	30	27	1.95E-03	small nuclear ribonucleoprotein complex
GO:0019867	110	86	1.98E-03	outer membrane
GO:0005794	767	536	2.10E-03	Golgi apparatus
GO:0005852	14	14	2.45E-03	eukaryotic translation initiation factor 3 complex
GO:0005689	24	22	3.11E-03	U12-type spliceosomal complex
GO:0000792	41	35	3.38E-03	heterochromatin
GO:0016591	75	60	3.57E-03	DNA-directed RNA polymerase II, holoenzyme
GO:0071339	28	25	3.77E-03	MLL1 complex
GO:0044432	570	400	4.86E-03	endoplasmic reticulum part
GO:0005720	27	24	5.23E-03	nuclear heterochromatin
GO:0005788	73	58	5.52E-03	endoplasmic reticulum lumen
GO:0022624	12	12	5.79E-03	proteasome accessory complex
GO:0030867	12	12	5.79E-03	rough endoplasmic reticulum membrane

GO:0030897	12	12	5.79E-03	HOPS complex
GO:0045263	12	12	5.79E-03	proton-transporting ATP synthase complex, coupling factor F(o)
GO:0044431	468	330	6.53E-03	Golgi apparatus part
GO:0000315	17	16	6.84E-03	organellar large ribosomal subunit
GO:0005762	17	16	6.84E-03	mitochondrial large ribosomal subunit
GO:0005839	17	16	6.84E-03	proteasome core complex
GO:0030135	147	110	7.09E-03	coated vesicle
GO:0044452	26	23	7.21E-03	nucleolar part
GO:0005758	34	29	7.87E-03	mitochondrial intermembrane space
GO:0005783	816	563	8.24E-03	endoplasmic reticulum
GO:0000151	136	102	8.33E-03	ubiquitin ligase complex
GO:0000782	11	11	8.89E-03	telomere cap complex
GO:0000783	11	11	8.89E-03	nuclear telomere cap complex
GO:0005744	11	11	8.89E-03	mitochondrial inner membrane presequence translocase complex

----

CE.2) Consistently Expressed Genes: Biological Process

GOID	GO #gene	CE #gene	p-value	GOterm
GO:0006412	394	364	1.36E-37	translation
GO:0006396	573	496	6.45E-32	RNA processing
GO:0044267	2196	1654	2.78E-29	cellular protein metabolic process
GO:0016071	472	410	3.77E-27	mRNA metabolic process
GO:0044237	6144	4271	6.03E-27	cellular metabolic process
GO:0008380	286	259	1.74E-23	RNA splicing
GO:0044260	4719	3323	4.31E-23	cellular macromolecule metabolic process
GO:0046907	694	562	9.65E-21	intracellular transport
GO:0019538	2435	1781	1.20E-20	protein metabolic process
GO:0006397	351	305	1.86E-20	mRNA processing
GO:0008152	6686	4577	4.21E-19	metabolic process
GO:0044265	458	382	1.89E-18	cellular macromolecule catabolic process
GO:0009057	546	447	2.56E-18	macromolecule catabolic process
GO:0000375	167	156	6.26E-18	RNA splicing, via transesterification reactions
GO:0043170	4997	3471	3.13E-17	macromolecule metabolic process
GO:0000377	162	151	3.83E-17	RNA splicing, via transesterification reactions with bulged adenosine as nucleophile
GO:0000398	162	151	3.83E-17	nuclear mRNA splicing, via spliceosome
GO:0010467	3073	2192	8.65E-17	gene expression
GO:0044238	6083	4175	8.85E-17	primary metabolic process
GO:0022415	205	184	5.21E-16	viral reproductive process
GO:0019080	137	129	7.85E-16	viral genome expression
GO:0019083	137	129	7.85E-16	viral transcription
GO:0010608	230	203	1.28E-15	posttranscriptional regulation of gene expression
GO:0016032	372	311	1.70E-15	viral reproduction
GO:0071843	205	183	2.38E-15	cellular component biogenesis at cellular level
GO:0022613	195	175	2.85E-15	ribonucleoprotein complex biogenesis
GO:0019058	184	166	4.52E-15	viral infectious cycle
GO:0022904	90	88	2.77E-14	respiratory electron transport chain
GO:0045184	807	622	6.72E-14	establishment of protein localization
GO:0034660	255	219	8.77E-14	ncRNA metabolic process
GO:0070647	375	309	1.08E-13	protein modification by small protein conjugation or removal
GO:0006414	105	100	1.75E-13	translational elongation
GO:0015031	790	608	2.23E-13	protein transport
GO:0090304	3154	2223	4.01E-13	nucleic acid metabolic process
GO:0032446	332	275	7.53E-13	protein modification by small protein conjugation

GO:0006417	144	131	7.85E-13	regulation of translation
GO:0034641	3973	2765	1.06E-12	cellular nitrogen compound metabolic process
GO:0045333	122	113	1.32E-12	cellular respiration
GO:0006415	93	89	1.66E-12	translational termination
GO:0051246	579	454	2.04E-12	regulation of protein metabolic process
GO:0016567	311	258	2.80E-12	protein ubiquitination
GO:0008104	954	719	3.09E-12	protein localization
GO:0019941	290	242	3.82E-12	modification-dependent protein catabolic process
GO:0043632	290	242	3.82E-12	modification-dependent macromolecule catabolic process
GO:0044248	1055	788	6.92E-12	cellular catabolic process
GO:0006807	4032	2797	9.09E-12	nitrogen compound metabolic process
GO:0009056	1225	905	1.14E-11	catabolic process
GO:0006511	285	237	1.34E-11	ubiquitin-dependent protein catabolic process
GO:0006139	3705	2578	1.82E-11	nucleobase, nucleoside, nucleotide and nucleic acid metabolic process
GO:0030163	348	283	2.52E-11	protein catabolic process
GO:0071841	2179	1555	2.56E-11	cellular component organization or biogenesis at cellular level
GO:0006996	1389	1016	2.94E-11	organelle organization
GO:0051603	297	245	3.31E-11	proteolysis involved in cellular protein catabolic process
GO:0006403	117	107	4.53E-11	RNA localization
GO:0033036	1143	845	4.97E-11	macromolecule localization
GO:0006405	64	63	5.44E-11	RNA export from nucleus
GO:0050657	116	106	6.37E-11	nucleic acid transport
GO:0050658	116	106	6.37E-11	RNA transport
GO:0051236	116	106	6.37E-11	establishment of RNA localization
GO:0044257	302	248	6.38E-11	cellular protein catabolic process
GO:0051168	89	84	7.17E-11	nuclear export
GO:0022900	119	108	1.24E-10	electron transport chain
GO:0042254	128	115	1.55E-10	ribosome biogenesis
GO:0043933	753	570	2.12E-10	macromolecular complex subunit organization
GO:0034621	517	402	3.25E-10	cellular macromolecular complex subunit organization
GO:0006119	51	51	3.96E-10	oxidative phosphorylation
GO:0044249	3515	2441	4.20E-10	cellular biosynthetic process
GO:0032268	519	403	4.29E-10	regulation of cellular protein metabolic process
GO:0006974	455	357	4.63E-10	response to DNA damage stimulus
GO:0033554	727	550	5.34E-10	cellular response to stress
GO:0071840	2678	1881	6.58E-10	cellular component organization or biogenesis
GO:0051028	104	95	6.80E-10	mRNA transport
GO:0048193	137	121	7.32E-10	Golgi vesicle transport
GO:0015931	132	117	8.39E-10	nucleobase, nucleoside, nucleotide and nucleic acid transport
GO:0009058	3572	2476	9.25E-10	biosynthetic process
GO:0006413	64	62	9.40E-10	translational initiation
GO:0006886	386	306	1.09E-09	intracellular protein transport
GO:0006913	215	180	1.36E-09	nucleocytoplasmic transport
GO:0043161	174	149	1.38E-09	proteasomal ubiquitin-dependent protein catabolic process
GO:0034470	186	158	1.58E-09	ncRNA processing
GO:0006406	55	54	2.18E-09	mRNA export from nucleus
GO:0051169	217	181	2.26E-09	nuclear transport
GO:0042773	46	46	3.33E-09	ATP synthesis coupled electron transport
GO:0042775	46	46	3.33E-09	mitochondrial ATP synthesis coupled electron transport
GO:0034645	2912	2031	4.38E-09	cellular macromolecule biosynthetic process
GO:0010498	178	151	4.46E-09	proteasomal protein catabolic process
GO:0071842	2088	1475	1.11E-08	cellular component organization at cellular level
GO:0009059	2945	2048	1.72E-08	macromolecule biosynthetic process
GO:0006457	191	159	2.85E-08	protein folding
GO:0044419	293	234	3.18E-08	interspecies interaction between organisms
GO:0071826	87	79	3.91E-08	ribonucleoprotein complex subunit organization
GO:0070727	485	370	8.83E-08	cellular macromolecule localization

GO:0016043	2577	1796	8.89E-08	cellular component organization
GO:0006364	94	84	9.00E-08	rRNA processing
GO:0042770	107	94	1.11E-07	signal transduction in response to DNA damage
GO:0034613	483	368	1.23E-07	cellular protein localization
GO:0051248	200	164	1.41E-07	negative regulation of protein metabolic process
GO:0071845	200	164	1.41E-07	cellular component disassembly at cellular level
GO:0016072	97	86	1.49E-07	rRNA metabolic process
GO:0007049	1000	727	1.69E-07	cell cycle
GO:0022618	82	74	2.04E-07	ribonucleoprotein complex assembly
GO:0006120	36	36	2.34E-07	mitochondrial electron transport, NADH to ubiquinone
GO:0016070	2266	1584	2.36E-07	RNA metabolic process
GO:0022411	201	164	2.72E-07	cellular component disassembly
GO:0032269	193	158	2.95E-07	negative regulation of cellular protein metabolic process
GO:0044085	995	722	3.06E-07	cellular component biogenesis
GO:0010605	775	570	3.42E-07	negative regulation of macromolecule metabolic process
GO:0016568	327	255	3.99E-07	chromatin modification
GO:0031123	75	68	4.17E-07	RNA 3'-end processing
GO:0051443	75	68	4.17E-07	positive regulation of ubiquitin-protein ligase activity
GO:0051436	65	60	4.17E-07	negative regulation of ubiquitin-protein ligase activity involved in mitotic cell cycle
GO:0006369	42	41	4.24E-07	termination of RNA polymerase II transcription
GO:0006446	42	41	4.24E-07	regulation of translational initiation
GO:0006521	48	46	4.88E-07	regulation of cellular amino acid metabolic process
GO:0071822	555	416	4.97E-07	protein complex subunit organization
GO:0006464	1552	1100	5.35E-07	protein modification process
GO:0043412	1620	1146	5.40E-07	macromolecule modification
GO:0006605	235	188	5.80E-07	protein targeting
GO:0031018	97	85	5.88E-07	endocrine pancreas development
GO:0043687	155	129	6.02E-07	post-translational protein modification
GO:0048610	264	209	6.08E-07	cellular process involved in reproduction
GO:0031145	78	70	7.48E-07	anaphase-promoting complex-dependent proteasomal ubiquitin-dependent protein catabolic process
GO:0006353	73	66	8.08E-07	transcription termination, DNA-dependent
GO:0051439	73	66	8.08E-07	regulation of ubiquitin-protein ligase activity involved in mitotic cell cycle
GO:0009987	8868	5879	8.28E-07	cellular process
GO:0006479	63	58	8.37E-07	protein methylation
GO:0008213	63	58	8.37E-07	protein alkylation
GO:0031571	63	58	8.37E-07	mitotic cell cycle G1/S transition DNA damage checkpoint
GO:0051437	68	62	8.42E-07	positive regulation of ubiquitin-protein ligase activity involved in mitotic cell cycle
GO:0051649	1011	730	9.14E-07	establishment of localization in cell
GO:0006520	301	235	9.67E-07	cellular amino acid metabolic process
GO:0051641	1121	805	9.97E-07	cellular localization
GO:0007005	133	112	1.00E-06	mitochondrion organization
GO:0034623	145	121	1.01E-06	cellular macromolecular complex disassembly
GO:0006977	57	53	1.11E-06	DNA damage response, signal transduction by p53 class mediator resulting in cell cycle arrest
GO:0072395	57	53	1.11E-06	signal transduction involved in cell cycle checkpoint
GO:0072401	57	53	1.11E-06	signal transduction involved in DNA integrity checkpoint
GO:0072404	57	53	1.11E-06	signal transduction involved in G1/S transition checkpoint
GO:0072413	57	53	1.11E-06	signal transduction involved in mitotic cell cycle checkpoint
GO:0072422	57	53	1.11E-06	signal transduction involved in DNA damage checkpoint
GO:0072431	57	53	1.11E-06	signal transduction involved in mitotic cell cycle G1/S transition DNA damage checkpoint
GO:0072474	57	53	1.11E-06	signal transduction involved in mitotic cell cycle G1/S checkpoint
GO:0031124	66	60	1.65E-06	mRNA 3'-end processing
GO:0051329	304	236	2.04E-06	interphase of mitotic cell cycle

GO:0007093	118	100	2.09E-06	mitotic cell cycle checkpoint
GO:0032984	146	121	2.10E-06	macromolecular complex disassembly
GO:0031400	134	112	2.18E-06	negative regulation of protein modification process
GO:0006259	538	401	2.22E-06	DNA metabolic process
GO:0030330	88	77	2.31E-06	DNA damage response, signal transduction by p53 class mediator
GO:0051726	491	368	2.35E-06	regulation of cell cycle
GO:2000045	74	66	2.68E-06	regulation of G1/S transition of mitotic cell cycle
GO:0006325	402	305	2.92E-06	chromatin organization
GO:0051325	309	239	2.92E-06	interphase
GO:0009892	815	592	3.22E-06	negative regulation of metabolic process
GO:0016570	182	147	3.55E-06	histone modification
GO:0000956	82	72	3.71E-06	nuclear-transcribed mRNA catabolic process
GO:0051352	68	61	4.12E-06	negative regulation of ligase activity
GO:0051444	68	61	4.12E-06	negative regulation of ubiquitin-protein ligase activity
GO:0051438	86	75	4.18E-06	regulation of ubiquitin-protein ligase activity
GO:0031397	81	71	5.01E-06	negative regulation of protein ubiquitination
GO:0006401	106	90	5.75E-06	RNA catabolic process
GO:0043624	134	111	5.81E-06	cellular protein complex disassembly
GO:0031016	110	93	5.89E-06	pancreas development
GO:0006915	968	695	6.33E-06	apoptosis
GO:0031324	764	555	6.58E-06	negative regulation of cellular metabolic process
GO:0031575	71	63	6.88E-06	mitotic cell cycle G1/S transition checkpoint
GO:0071779	71	63	6.88E-06	G1/S transition checkpoint
GO:0012501	975	699	8.21E-06	programmed cell death
GO:0045786	334	255	8.37E-06	negative regulation of cell cycle
GO:0016569	185	148	9.21E-06	covalent chromatin modification
GO:0008219	1063	758	1.13E-05	cell death
GO:0043241	135	111	1.17E-05	protein complex disassembly
GO:0065003	620	454	1.26E-05	macromolecular complex assembly
GO:0018196	82	71	1.34E-05	peptidyl-asparagine modification
GO:0018279	82	71	1.34E-05	protein N-linked glycosylation via asparagine
GO:0006091	324	247	1.40E-05	generation of precursor metabolites and energy
GO:0016265	1064	758	1.40E-05	death
GO:0051276	531	392	1.43E-05	chromosome organization
GO:0006402	89	76	2.00E-05	mRNA catabolic process
GO:0000077	101	85	2.16E-05	DNA damage checkpoint
GO:0042180	595	435	2.49E-05	cellular ketone metabolic process
GO:0044106	350	264	2.89E-05	cellular amine metabolic process
GO:0033238	57	51	3.15E-05	regulation of cellular amine metabolic process
GO:0051351	79	68	3.15E-05	positive regulation of ligase activity
GO:0000082	157	126	3.17E-05	G1/S transition of mitotic cell cycle
GO:0006487	87	74	3.46E-05	protein N-linked glycosylation
GO:0031396	130	106	3.74E-05	regulation of protein ubiquitination
GO:0019752	581	424	4.20E-05	carboxylic acid metabolic process
GO:0043436	581	424	4.20E-05	oxoacid metabolic process
GO:0006281	317	240	4.29E-05	DNA repair
GO:0015980	234	181	4.60E-05	energy derivation by oxidation of organic compounds
GO:0031398	102	85	4.64E-05	positive regulation of protein ubiquitination
GO:0000075	195	153	4.72E-05	cell cycle checkpoint
GO:0000387	30	29	5.05E-05	spliceosomal snRNP assembly
GO:0071158	60	53	5.12E-05	positive regulation of cell cycle arrest
GO:0051247	278	212	5.44E-05	positive regulation of protein metabolic process
GO:0043414	89	75	6.01E-05	macromolecule methylation
GO:0051186	200	156	7.03E-05	cofactor metabolic process
GO:0000278	599	435	7.19E-05	mitotic cell cycle
GO:0016571	45	41	7.22E-05	histone methylation
GO:0032270	262	200	7.91E-05	positive regulation of cellular protein metabolic process

GO:0006399	119	97	8.16E-05	tRNA metabolic process
GO:0018193	339	254	9.05E-05	peptidyl-amino acid modification
GO:0006839	75	64	9.60E-05	mitochondrial transport
GO:0007050	288	218	9.74E-05	cell cycle arrest
GO:0034622	385	286	9.81E-05	cellular macromolecular complex assembly
GO:0007346	219	169	1.02E-04	regulation of mitotic cell cycle
GO:0033365	236	181	1.09E-04	protein localization to organelle
GO:0070936	28	27	1.11E-04	protein K48-linked ubiquitination
GO:0046782	53	47	1.11E-04	regulation of viral transcription
GO:0072594	66	57	1.14E-04	establishment of protein localization to organelle
GO:0048524	57	50	1.28E-04	positive regulation of viral reproduction
GO:0006418	48	43	1.28E-04	tRNA aminoacylation for protein translation
GO:0043038	48	43	1.28E-04	amino acid activation
GO:0043039	48	43	1.28E-04	tRNA aminoacylation
GO:0051340	90	75	1.30E-04	regulation of ligase activity
GO:0048523	1626	1129	1.30E-04	negative regulation of cellular process
GO:0010629	552	401	1.32E-04	negative regulation of gene expression
GO:0006082	590	427	1.39E-04	organic acid metabolic process
GO:0006888	43	39	1.41E-04	ER to Golgi vesicle-mediated transport
GO:0016197	77	65	1.68E-04	endosome transport
GO:0016458	60	52	1.89E-04	gene silencing
GO:0032434	32	30	2.01E-04	regulation of proteasomal ubiquitin-dependent protein catabolic process
GO:0071156	201	155	2.08E-04	regulation of cell cycle arrest
GO:0031570	107	87	2.24E-04	DNA integrity checkpoint
GO:0006308	55	48	2.32E-04	DNA catabolic process
GO:0000084	118	95	2.36E-04	S phase of mitotic cell cycle
GO:0008334	26	25	2.41E-04	histone mRNA metabolic process
GO:0006352	99	81	2.46E-04	transcription initiation, DNA-dependent
GO:0035270	125	100	2.57E-04	endocrine system development
GO:0043067	739	527	2.70E-04	regulation of programmed cell death
GO:0010604	920	650	2.76E-04	positive regulation of macromolecule metabolic process
GO:0042981	732	522	2.89E-04	regulation of apoptosis
GO:0032259	98	80	3.10E-04	methylation
GO:0050434	49	43	3.80E-04	positive regulation of viral transcription
GO:0051320	123	98	3.91E-04	S phase
GO:2000113	574	413	3.98E-04	negative regulation of cellular macromolecule biosynthetic process
GO:0034968	35	32	4.14E-04	histone lysine methylation
GO:0006260	207	158	4.37E-04	DNA replication
GO:0010941	749	532	4.65E-04	regulation of cell death
GO:0042147	24	23	5.24E-04	retrograde transport, endosome to Golgi
GO:0010564	280	209	5.32E-04	regulation of cell cycle process
GO:0022402	763	541	5.41E-04	cell cycle process
GO:0006626	34	31	5.82E-04	protein targeting to mitochondrion
GO:0006903	29	27	5.95E-04	vesicle targeting
GO:0048519	1743	1200	6.13E-04	negative regulation of biological process
GO:0022403	636	454	6.27E-04	cell cycle phase
GO:0061136	38	34	7.27E-04	regulation of proteasomal protein catabolic process
GO:0051568	17	17	7.46E-04	histone H3-K4 methylation
GO:0006900	23	22	7.70E-04	membrane budding
GO:0006901	23	22	7.70E-04	vesicle coating
GO:2000243	59	50	8.01E-04	positive regulation of reproductive process
GO:0050792	63	53	8.07E-04	regulation of viral reproduction
GO:0009060	33	30	8.16E-04	aerobic respiration
GO:0022607	873	614	8.42E-04	cellular component assembly
GO:0051172	560	401	8.83E-04	negative regulation of nitrogen compound metabolic process
GO:0000216	74	61	9.81E-04	M/G1 transition of mitotic cell cycle

GO:0009312	37	33	1.00E-03	oligosaccharide biosynthetic process
GO:0015985	37	33	1.00E-03	energy coupled proton transport, down electrochemical gradient
GO:0015986	37	33	1.00E-03	ATP synthesis coupled proton transport
GO:0042274	16	16	1.14E-03	ribosomal small subunit biogenesis
GO:0017038	156	120	1.24E-03	protein import
GO:0045934	556	397	1.32E-03	negative regulation of nucleobase, nucleoside, nucleotide and nucleic acid metabolic process
GO:0006368	65	54	1.33E-03	transcription elongation from RNA polymerase II promoter
GO:0031647	53	45	1.35E-03	regulation of protein stability
GO:2000241	84	68	1.36E-03	regulation of reproductive process
GO:0045454	57	48	1.36E-03	cell redox homeostasis
GO:0070585	36	32	1.38E-03	protein localization in mitochondrion
GO:0006367	72	59	1.58E-03	transcription initiation from RNA polymerase II promoter
GO:0030518	72	59	1.58E-03	steroid hormone receptor signaling pathway
GO:0006488	31	28	1.59E-03	dolichol-linked oligosaccharide biosynthetic process
GO:0034976	31	28	1.59E-03	response to endoplasmic reticulum stress
GO:0048199	21	20	1.65E-03	vesicle targeting, to, from or within Golgi
GO:0009109	26	24	1.73E-03	coenzyme catabolic process
GO:0009067	15	15	1.74E-03	aspartate family amino acid biosynthetic process
GO:0006366	808	567	1.83E-03	transcription from RNA polymerase II promoter
GO:0072655	35	31	1.88E-03	establishment of protein localization in mitochondrion
GO:0006730	136	105	1.97E-03	one-carbon metabolic process
GO:0016481	504	360	2.10E-03	negative regulation of transcription
GO:0051188	118	92	2.14E-03	cofactor biosynthetic process
GO:0031325	947	660	2.22E-03	positive regulation of cellular metabolic process
GO:0010558	588	417	2.26E-03	negative regulation of macromolecule biosynthetic process
GO:0006461	446	320	2.33E-03	protein complex assembly
GO:0070271	449	322	2.38E-03	protein complex biogenesis
GO:0009308	398	287	2.41E-03	amine metabolic process
GO:0006891	20	19	2.42E-03	intra-Golgi vesicle-mediated transport
GO:0030522	85	68	2.45E-03	intracellular receptor mediated signaling pathway
GO:0007006	25	23	2.45E-03	mitochondrial membrane organization
GO:0046356	25	23	2.45E-03	acetyl-CoA catabolic process
GO:0070979	25	23	2.45E-03	protein K11-linked ubiquitination
GO:0006475	70	57	2.53E-03	internal protein amino acid acetylation
GO:0018394	70	57	2.53E-03	peptidyl-lysine acetylation
GO:0043648	34	30	2.57E-03	dicarboxylic acid metabolic process
GO:0045947	14	14	2.66E-03	negative regulation of translational initiation
GO:0006986	58	48	2.89E-03	response to unfolded protein
GO:0015992	50	42	3.02E-03	proton transport
GO:0045727	29	26	3.07E-03	positive regulation of translation
GO:0018393	69	56	3.19E-03	internal peptidyl-lysine acetylation
GO:0006732	157	119	3.30E-03	coenzyme metabolic process
GO:0031399	331	240	3.35E-03	regulation of protein modification process
GO:0006099	24	22	3.47E-03	tricarboxylic acid cycle
GO:0006779	24	22	3.47E-03	porphyrin biosynthetic process
GO:0033014	24	22	3.47E-03	tetrapyrrole biosynthetic process
GO:0000245	33	29	3.50E-03	spliceosome assembly
GO:0006473	76	61	3.51E-03	protein acetylation
GO:0070534	19	18	3.52E-03	protein K63-linked ubiquitination
GO:0009893	983	682	3.65E-03	positive regulation of metabolic process
GO:0006892	57	47	3.70E-03	post-Golgi vesicle-mediated transport
GO:0046364	57	47	3.70E-03	monosaccharide biosynthetic process
GO:0031327	601	424	3.78E-03	negative regulation of cellular biosynthetic process
GO:0044281	1542	1056	3.80E-03	small molecule metabolic process
GO:0055114	669	470	3.86E-03	oxidation-reduction process
GO:0016573	68	55	4.00E-03	histone acetylation
GO:0042176	79	63	4.04E-03	regulation of protein catabolic process

GO:0042776	13	13	4.06E-03	mitochondrial ATP synthesis coupled proton transport
GO:0071844	658	462	4.45E-03	cellular component assembly at cellular level
GO:0009890	608	428	4.61E-03	negative regulation of biosynthetic process
GO:0060255	2704	1826	4.78E-03	regulation of macromolecule metabolic process
GO:0006890	23	21	4.90E-03	retrograde vesicle-mediated transport, Golgi to ER
GO:0000288	52	43	4.91E-03	nuclear-transcribed mRNA catabolic process, deadenylation-dependent decay
GO:0006818	52	43	4.91E-03	hydrogen transport
GO:0009311	52	43	4.91E-03	oligosaccharide metabolic process
GO:0006612	36	31	5.04E-03	protein targeting to membrane
GO:0045862	36	31	5.04E-03	positive regulation of proteolysis
GO:0030968	18	17	5.13E-03	endoplasmic reticulum unfolded protein response
GO:0034620	18	17	5.13E-03	cellular response to unfolded protein
GO:0016192	594	418	5.27E-03	vesicle-mediated transport
GO:0006354	92	72	5.33E-03	transcription elongation, DNA-dependent
GO:0009142	81	64	5.65E-03	nucleoside triphosphate biosynthetic process
GO:0042558	27	24	5.84E-03	pteridine-containing compound metabolic process
GO:0060548	329	237	5.99E-03	negative regulation of cell death
GO:0006921	55	45	5.99E-03	cellular component disassembly involved in apoptosis
GO:0043066	316	228	6.14E-03	negative regulation of apoptosis
GO:0006613	12	12	6.21E-03	cotranslational protein targeting to membrane
GO:0043069	319	230	6.27E-03	negative regulation of programmed cell death
GO:0006458	51	42	6.29E-03	de novo' protein folding
GO:0007034	31	27	6.41E-03	vacuolar transport
GO:0051187	31	27	6.41E-03	cofactor catabolic process
GO:0051052	105	81	6.45E-03	regulation of DNA metabolic process
GO:0007569	39	33	6.78E-03	cell aging
GO:0090305	39	33	6.78E-03	nucleic acid phosphodiester bond hydrolysis
GO:0018205	87	68	7.06E-03	peptidyl-lysine modification
GO:0006810	2142	1451	7.14E-03	transport
GO:0006007	58	47	7.15E-03	glucose catabolic process
GO:0031401	208	153	7.35E-03	positive regulation of protein modification process
GO:0043543	83	65	7.72E-03	protein acylation
GO:0051170	114	87	8.14E-03	nuclear import
GO:0051084	46	38	8.39E-03	de novo' posttranslational protein folding
GO:0009145	79	62	8.44E-03	purine nucleoside triphosphate biosynthetic process
GO:0009201	79	62	8.44E-03	ribonucleoside triphosphate biosynthetic process
GO:0051234	2180	1475	8.45E-03	establishment of localization
GO:0006916	190	140	9.12E-03	anti-apoptosis
GO:0000097	11	11	9.49E-03	sulfur amino acid biosynthetic process
GO:0006378	11	11	9.49E-03	mRNA polyadenylation
GO:0042273	11	11	9.49E-03	ribosomal large subunit biogenesis
GO:0043984	11	11	9.49E-03	histone H4-K16 acetylation
GO:0045815	11	11	9.49E-03	positive regulation of gene expression, epigenetic
GO:0010557	676	471	9.55E-03	positive regulation of macromolecule biosynthetic process
GO:0000718	21	19	9.66E-03	nucleotide-excision repair, DNA damage removal
GO:0030262	21	19	9.66E-03	apoptotic nuclear change
GO:0032436	21	19	9.66E-03	positive regulation of proteasomal ubiquitin-dependent protein catabolic process

----

CE.3) Consistently Expressed Genes: Molecular Function

GOID	GO #gene	CE #gene	p-value	GOterm

GO:0005515	5353	3840	2.74E-45	protein binding
GO:0003723	729	609	9.16E-30	RNA binding
GO:0003735	155	150	4.54E-22	structural constituent of ribosome
GO:0005488	8579	5731	1.75E-11	binding
GO:0051082	110	98	9.42E-09	unfolded protein binding
GO:0016874	401	312	2.32E-08	ligase activity
GO:0003954	37	37	1.43E-07	NADH dehydrogenase activity
GO:0008137	37	37	1.43E-07	NADH dehydrogenase (ubiquinone) activity
GO:0050136	37	37	1.43E-07	NADH dehydrogenase (quinone) activity
GO:0003743	50	48	2.08E-07	translation initiation factor activity
GO:0016655	42	41	3.95E-07	oxidoreductase activity, acting on NADH or NADPH, quinone or similar compound as acceptor
GO:0016879	267	209	2.29E-06	ligase activity, forming carbon-nitrogen bonds
GO:0019787	209	167	2.42E-06	small conjugating protein ligase activity
GO:0016881	238	188	2.47E-06	acid-amino acid ligase activity
GO:0000166	1863	1299	6.24E-06	nucleotide binding
GO:0004842	189	151	7.42E-06	ubiquitin-protein ligase activity
GO:0051287	40	38	9.66E-06	NAD+ or NADH binding
GO:0003713	198	156	2.29E-05	transcription coactivator activity
GO:0003729	61	54	3.48E-05	mRNA binding
GO:0016651	69	60	4.43E-05	oxidoreductase activity, acting on NADH or NADPH
GO:0003712	325	245	4.99E-05	transcription cofactor activity
GO:0008135	93	78	5.50E-05	translation factor activity, nucleic acid binding
GO:0015078	80	68	6.93E-05	hydrogen ion transmembrane transporter activity
GO:0000988	326	245	7.15E-05	protein binding transcription factor activity
GO:0000989	326	245	7.15E-05	transcription factor binding transcription factor activity
GO:0004812	49	44	8.67E-05	aminoacyl-tRNA ligase activity
GO:0016875	49	44	8.67E-05	ligase activity, forming carbon-oxygen bonds
GO:0016876	49	44	8.67E-05	ligase activity, forming aminoacyl-tRNA and related compounds
GO:0008565	79	67	9.09E-05	protein transporter activity
GO:0008276	61	53	1.31E-04	protein methyltransferase activity
GO:0003824	4014	2709	1.68E-04	catalytic activity
GO:0043021	42	38	1.86E-04	ribonucleoprotein binding
GO:0048037	199	152	4.67E-04	cofactor binding
GO:0005198	426	310	5.20E-04	structural molecule activity
GO:0003676	2475	1684	7.21E-04	nucleic acid binding
GO:0003697	55	47	7.35E-04	single-stranded DNA binding
GO:0042054	46	40	8.82E-04	histone methyltransferase activity
GO:0008134	242	181	9.14E-04	transcription factor binding
GO:0035257	74	61	9.14E-04	nuclear hormone receptor binding
GO:0051427	81	66	1.06E-03	hormone receptor binding
GO:0002039	32	29	1.09E-03	p53 binding
GO:0050662	149	115	1.13E-03	coenzyme binding
GO:0016853	117	92	1.20E-03	isomerase activity
GO:0031072	72	59	1.48E-03	heat shock protein binding
GO:0019902	48	41	1.65E-03	phosphatase binding
GO:0016278	39	34	1.99E-03	lysine N-methyltransferase activity
GO:0016279	39	34	1.99E-03	protein-lysine N-methyltransferase activity
GO:0016741	166	126	2.10E-03	transferase activity, transferring one-carbon groups
GO:0019843	25	23	2.37E-03	rRNA binding
GO:0008320	14	14	2.59E-03	protein transmembrane transporter activity
GO:0022884	14	14	2.59E-03	macromolecule transmembrane transporter activity
GO:0016491	503	358	2.65E-03	oxidoreductase activity
GO:0018024	38	33	2.69E-03	histone-lysine N-methyltransferase activity
GO:0019903	42	36	2.82E-03	protein phosphatase binding
GO:0008168	163	123	3.39E-03	methyltransferase activity
GO:0004129	19	18	3.42E-03	cytochrome-c oxidase activity
GO:0015002	19	18	3.42E-03	heme-copper terminal oxidase activity

GO:0016675	19	18	3.42E-03	oxidoreductase activity, acting on a heme group of donors
GO:0016676	19	18	3.42E-03	oxidoreductase activity, acting on a heme group of donors, oxygen as acceptor
GO:0008026	100	78	4.15E-03	ATP-dependent helicase activity
GO:0070035	100	78	4.15E-03	purine NTP-dependent helicase activity
GO:0008757	89	70	4.46E-03	S-adenosylmethionine-dependent methyltransferase activity
GO:0016859	32	28	4.56E-03	cis-trans isomerase activity
GO:0047485	67	54	4.72E-03	protein N-terminus binding
GO:0031625	74	59	5.07E-03	ubiquitin protein ligase binding
GO:0003725	27	24	5.64E-03	double-stranded RNA binding
GO:0003755	31	27	6.18E-03	peptidyl-prolyl cis-trans isomerase activity
GO:0042393	62	50	6.34E-03	histone binding
GO:0032182	39	33	6.50E-03	small conjugating protein binding
GO:0008170	58	47	6.78E-03	N-methyltransferase activity
GO:0004298	17	16	7.24E-03	threonine-type endopeptidase activity
GO:0008601	17	16	7.24E-03	protein phosphatase type 2A regulator activity
GO:0070003	17	16	7.24E-03	threonine-type peptidase activity
GO:0003724	26	23	7.75E-03	RNA helicase activity
GO:0046966	26	23	7.75E-03	thyroid hormone receptor binding
GO:0008536	11	11	9.29E-03	Ran GTPase binding
GO:0004386	133	100	9.34E-03	helicase activity
GO:0005048	21	19	9.38E-03	signal sequence binding

Part II. Inconsistently Expressed Genes

IE.1) Inconsistently Expressed Genes: Cellular Component

GOID	GO #gene	CE #gene	p-value	GOterm
GO:0005576	810	461	1.83E-40	extracellular region
GO:0071944	2199	1004	9.88E-32	cell periphery
GO:0005886	2160	988	1.63E-31	plasma membrane
GO:0031224	2972	1293	1.80E-30	intrinsic to membrane
GO:0016021	2927	1264	7.74E-28	integral to membrane
GO:0044421	424	253	2.46E-26	extracellular region part
GO:0044425	3626	1485	7.48E-21	membrane part
GO:0044459	1214	571	1.51E-20	plasma membrane part
GO:0031226	617	318	1.96E-18	intrinsic to plasma membrane
GO:0005887	603	310	8.78E-18	integral to plasma membrane
GO:0005615	306	176	2.21E-16	extracellular space
GO:0031012	180	114	4.52E-15	extracellular matrix
GO:0005578	153	100	1.11E-14	proteinaceous extracellular matrix
GO:0016020	4585	1760	4.56E-11	membrane
GO:0034702	95	62	1.39E-09	ion channel complex
GO:0044456	165	91	6.59E-08	synapse part
GO:0045211	81	52	6.67E-08	postsynaptic membrane
GO:0009986	219	112	4.57E-07	cell surface
GO:0009897	74	46	1.47E-06	external side of plasma membrane
GO:0045202	238	118	1.77E-06	synapse
GO:0034703	66	41	5.57E-06	cation channel complex
GO:0044420	67	41	9.54E-06	extracellular matrix part
GO:0005604	48	30	8.52E-05	basement membrane
GO:0042995	555	234	1.59E-04	cell projection
GO:0044463	263	120	1.77E-04	cell projection part
GO:0005892	8	8	2.18E-04	nicotinic acetylcholine-gated receptor-channel complex
GO:0034706	8	8	2.18E-04	sodium channel complex
GO:0035085	28	19	3.80E-04	cilium axoneme
GO:0031225	54	31	5.77E-04	anchored to membrane
GO:0044442	7	7	6.27E-04	microtubule-based flagellum part
GO:0044460	7	7	6.27E-04	flagellum part
GO:0005930	33	21	7.00E-04	axoneme
GO:0044441	46	27	8.06E-04	cilium part
GO:0032982	9	8	1.36E-03	myosin filament
GO:0005929	115	56	1.51E-03	cilium
GO:0031232	6	6	1.80E-03	extrinsic to external side of plasma membrane
GO:0001533	11	9	1.98E-03	cornified envelope
GO:0009434	11	9	1.98E-03	microtubule-based flagellum
GO:0016010	13	10	2.43E-03	dystrophin-associated glycoprotein complex
GO:0034704	17	12	2.90E-03	calcium channel complex
GO:0005859	10	8	4.70E-03	muscle myosin complex
GO:0016460	10	8	4.70E-03	myosin II complex
GO:0001518	5	5	5.16E-03	voltage-gated sodium channel complex
GO:0032279	5	5	5.16E-03	asymmetric synapse
GO:0042613	5	5	5.16E-03	MHC class II protein complex
GO:0042383	40	22	7.13E-03	sarcolemma
GO:0043292	80	39	7.18E-03	contractile fiber
GO:0030313	7	6	8.83E-03	cell envelope
GO:0030016	76	37	8.93E-03	myofibril
GO:0043005	254	107	9.23E-03	neuron projection

GO:0042734	23	14	9.67E-03	presynaptic membrane
GO:0044449	74	36	9.96E-03	contractile fiber part

IE.2) Inconsistently Expressed Genes: Biological Process

GOID	GO #gene	CE #gene	p-value	GOterm
GO:0003008	735	384	8.40E-25	system process
GO:0032501	2926	1201	8.04E-19	multicellular organismal process
GO:0050877	524	266	2.94E-15	neurological system process
GO:0007267	443	225	4.35E-13	cell-cell signaling
GO:0007600	232	131	3.78E-12	sensory perception
GO:0007186	230	130	4.16E-12	G-protein coupled receptor protein signaling pathway
GO:0007606	44	37	1.57E-11	sensory perception of chemical stimulus
GO:0007608	36	32	1.73E-11	sensory perception of smell
GO:0007155	517	250	1.88E-11	cell adhesion
GO:0022610	517	250	1.88E-11	biological adhesion
GO:0023052	2556	1016	7.31E-11	signaling
GO:0006811	506	238	1.62E-09	ion transport
GO:0007166	1151	488	2.01E-09	cell surface receptor linked signaling pathway
GO:0007268	249	128	2.18E-08	synaptic transmission
GO:0006936	118	70	2.70E-08	muscle contraction
GO:0009190	57	40	3.98E-08	cyclic nucleotide biosynthetic process
GO:0019226	283	141	5.46E-08	transmission of nerve impulse
GO:0035637	283	141	5.46E-08	multicellular organismal signaling
GO:0003012	127	73	8.47E-08	muscle system process
GO:0050896	3789	1427	1.18E-07	response to stimulus
GO:0007165	2343	913	1.34E-07	signal transduction
GO:0006954	171	92	1.43E-07	inflammatory response
GO:0009187	71	46	1.74E-07	cyclic nucleotide metabolic process
GO:0044057	157	85	2.96E-07	regulation of system process
GO:0046058	56	38	3.49E-07	cAMP metabolic process
GO:0007154	1045	434	3.53E-07	cell communication
GO:0040011	581	257	3.54E-07	locomotion
GO:0009605	598	263	4.64E-07	response to external stimulus
GO:0032101	109	63	4.92E-07	regulation of response to external stimulus
GO:0003013	127	71	5.55E-07	circulatory system process
GO:0008015	127	71	5.55E-07	blood circulation
GO:0030802	53	36	6.84E-07	regulation of cyclic nucleotide biosynthetic process
GO:0030808	53	36	6.84E-07	regulation of nucleotide biosynthetic process
GO:0030154	1294	524	8.61E-07	cell differentiation
GO:0030001	301	144	8.79E-07	metal ion transport
GO:0048869	1330	536	1.28E-06	cellular developmental process
GO:0006171	50	34	1.34E-06	cAMP biosynthetic process
GO:0009653	961	397	2.29E-06	anatomical structure morphogenesis
GO:0009611	556	243	2.32E-06	response to wounding
GO:0007586	43	30	2.47E-06	digestion
GO:0030799	57	37	2.61E-06	regulation of cyclic nucleotide metabolic process
GO:0007588	34	25	3.81E-06	excretion
GO:0006935	305	143	3.92E-06	chemotaxis
GO:0042330	305	143	3.92E-06	taxis
GO:0009124	81	48	4.25E-06	nucleoside monophosphate biosynthetic process
GO:0030817	48	32	5.32E-06	regulation of cAMP biosynthetic process
GO:0048514	180	91	5.76E-06	blood vessel morphogenesis
GO:0046903	400	180	6.00E-06	secretion

GO:0007275	2137	823	6.80E-06	multicellular organismal development
GO:0007399	906	373	7.04E-06	nervous system development
GO:0030804	17	15	7.16E-06	positive regulation of cyclic nucleotide biosynthetic process
GO:0030810	17	15	7.16E-06	positive regulation of nucleotide biosynthetic process
GO:0051239	707	297	1.02E-05	regulation of multicellular organismal process
GO:0006812	355	161	1.09E-05	cation transport
GO:0030801	19	16	1.18E-05	positive regulation of cyclic nucleotide metabolic process
GO:0045981	19	16	1.18E-05	positive regulation of nucleotide metabolic process
GO:0006939	26	20	1.19E-05	smooth muscle contraction
GO:0048646	343	156	1.23E-05	anatomical structure formation involved in morphogenesis
GO:0030814	50	32	1.91E-05	regulation of cAMP metabolic process
GO:0009123	100	55	1.95E-05	nucleoside monophosphate metabolic process
GO:0048731	1777	688	2.35E-05	system development
GO:0017144	13	12	2.49E-05	drug metabolic process
GO:0030816	13	12	2.49E-05	positive regulation of cAMP metabolic process
GO:0030819	13	12	2.49E-05	positive regulation of cAMP biosynthetic process
GO:0048468	670	280	2.94E-05	cell development
GO:0009888	549	234	2.96E-05	tissue development
GO:0010817	179	88	3.25E-05	regulation of hormone levels
GO:0007204	51	32	3.44E-05	elevation of cytosolic calcium ion concentration
GO:0051046	165	82	3.66E-05	regulation of secretion
GO:0006874	86	48	3.91E-05	cellular calcium ion homeostasis
GO:0006140	187	91	3.94E-05	regulation of nucleotide metabolic process
GO:0031279	43	28	3.99E-05	regulation of cyclase activity
GO:0001525	151	76	4.02E-05	angiogenesis
GO:0007188	37	25	4.09E-05	G-protein signaling, coupled to cAMP nucleotide second messenger
GO:0007218	39	26	4.10E-05	neuropeptide signaling pathway
GO:0072503	93	51	4.31E-05	cellular divalent inorganic cation homeostasis
GO:0045165	80	45	5.23E-05	cell fate commitment
GO:0048856	1968	753	5.44E-05	anatomical structure development
GO:0065007	5494	1987	5.52E-05	biological regulation
GO:0048870	369	163	5.58E-05	cell motility
GO:0051674	369	163	5.58E-05	localization of cell
GO:0048871	54	33	5.64E-05	multicellular organismal homeostasis
GO:0007389	191	92	5.85E-05	pattern specification process
GO:0050806	12	11	6.71E-05	positive regulation of synaptic transmission
GO:0051971	12	11	6.71E-05	positive regulation of transmission of nerve impulse
GO:0003001	153	76	7.09E-05	generation of a signal involved in cell-cell signaling
GO:0023061	153	76	7.09E-05	signal release
GO:0051339	44	28	7.36E-05	regulation of lyase activity
GO:0045761	42	27	7.64E-05	regulation of adenylate cyclase activity
GO:0006816	90	49	7.73E-05	calcium ion transport
GO:0055074	90	49	7.73E-05	calcium ion homeostasis
GO:0032502	2362	892	7.89E-05	developmental process
GO:0032102	38	25	8.04E-05	negative regulation of response to external stimulus
GO:0072507	97	52	8.15E-05	divalent inorganic cation homeostasis
GO:0030182	500	212	1.02E-04	neuron differentiation
GO:0006952	459	196	1.16E-04	defense response
GO:0019933	49	30	1.16E-04	cAMP-mediated signaling
GO:0008217	45	28	1.31E-04	regulation of blood pressure
GO:0048699	531	223	1.36E-04	generation of neurons
GO:0006813	78	43	1.43E-04	potassium ion transport
GO:0006814	78	43	1.43E-04	sodium ion transport
GO:0051480	56	33	1.54E-04	cytosolic calcium ion homeostasis
GO:0001944	225	104	1.58E-04	vasculature development
GO:0009566	35	23	1.59E-04	fertilization
GO:0007271	11	10	1.79E-04	synaptic transmission, cholinergic

GO:0007423	159	77	1.83E-04	sensory organ development
GO:0032940	335	147	1.86E-04	secretion by cell
GO:0007187	50	30	1.95E-04	G-protein signaling, coupled to cyclic nucleotide second messenger
GO:0016477	351	153	2.04E-04	cell migration
GO:0015672	189	89	2.09E-04	monovalent inorganic cation transport
GO:0032989	458	194	2.13E-04	cellular component morphogenesis
GO:0048878	349	152	2.24E-04	chemical homeostasis
GO:0010959	68	38	2.36E-04	regulation of metal ion transport
GO:0001568	212	98	2.40E-04	blood vessel development
GO:0000904	378	163	2.43E-04	cell morphogenesis involved in differentiation
GO:0001775	373	161	2.50E-04	cell activation
GO:0042471	42	26	2.56E-04	ear morphogenesis
GO:0042446	40	25	2.71E-04	hormone biosynthetic process
GO:0051048	38	24	2.85E-04	negative regulation of secretion
GO:0006955	442	187	2.97E-04	immune response
GO:0006937	36	23	2.98E-04	regulation of muscle contraction
GO:0042472	36	23	2.98E-04	inner ear morphogenesis
GO:0048666	424	180	3.12E-04	neuron development
GO:0007338	26	18	3.17E-04	single fertilization
GO:0003018	51	30	3.19E-04	vascular process in circulatory system
GO:0007416	30	20	3.22E-04	synapse assembly
GO:0019932	164	78	3.42E-04	second-messenger-mediated signaling
GO:0051240	164	78	3.42E-04	positive regulation of multicellular organismal process
GO:0050808	45	27	4.05E-04	synapse organization
GO:0000902	434	183	4.20E-04	cell morphogenesis
GO:0002683	56	32	4.21E-04	negative regulation of immune system process
GO:0048667	335	145	4.31E-04	cell morphogenesis involved in neuron differentiation
GO:0050801	263	117	4.32E-04	ion homeostasis
GO:0008016	43	26	4.36E-04	regulation of heart contraction
GO:0015718	43	26	4.36E-04	monocarboxylic acid transport
GO:0048812	338	146	4.60E-04	neuron projection morphogenesis
GO:0007417	302	132	4.74E-04	central nervous system development
GO:0042312	10	9	4.75E-04	regulation of vasodilation
GO:0002088	17	13	4.97E-04	lens development in camera-type eye
GO:0051924	52	30	5.06E-04	regulation of calcium ion transport
GO:0051716	2849	1054	5.16E-04	cellular response to stimulus
GO:0016337	206	94	5.51E-04	cell-cell adhesion
GO:0050866	35	22	5.62E-04	negative regulation of cell activation
GO:0048016	19	14	5.64E-04	inositol phosphate-mediated signaling
GO:0006355	1429	548	5.67E-04	regulation of transcription, DNA-dependent
GO:0055085	593	242	5.73E-04	transmembrane transport
GO:0045909	7	7	5.80E-04	positive regulation of vasodilation
GO:0015833	96	49	5.96E-04	peptide transport
GO:0016339	21	15	6.09E-04	calcium-dependent cell-cell adhesion
GO:0007194	27	18	6.45E-04	negative regulation of adenylate cyclase activity
GO:0007270	27	18	6.45E-04	nerve-nerve synaptic transmission
GO:0031280	27	18	6.45E-04	negative regulation of cyclase activity
GO:0051350	27	18	6.45E-04	negative regulation of lyase activity
GO:0061041	27	18	6.45E-04	regulation of wound healing
GO:0006928	440	184	6.62E-04	cellular component movement
GO:0048729	192	88	6.85E-04	tissue morphogenesis
GO:0009914	106	53	6.88E-04	hormone transport
GO:0007411	225	101	7.10E-04	axon guidance
GO:0009790	438	183	7.20E-04	embryo development
GO:0070661	71	38	7.45E-04	leukocyte proliferation
GO:0090257	42	25	7.79E-04	regulation of muscle system process
GO:0048839	53	30	7.85E-04	inner ear development

GO:0035150	40	24	8.43E-04	regulation of tube size
GO:0050880	40	24	8.43E-04	regulation of blood vessel size
GO:0046879	102	51	8.61E-04	hormone secretion
GO:0050954	60	33	8.72E-04	sensory perception of mechanical stimulus
GO:0051050	171	79	9.44E-04	positive regulation of transport
GO:0050727	49	28	9.51E-04	regulation of inflammatory response
GO:0031646	14	11	9.57E-04	positive regulation of neurological system process
GO:0035249	14	11	9.57E-04	synaptic transmission, glutamatergic
GO:0046849	14	11	9.57E-04	bone remodeling
GO:0019935	65	35	1.03E-03	cyclic-nucleotide-mediated signaling
GO:0007601	122	59	1.03E-03	visual perception
GO:0050953	122	59	1.03E-03	sensory perception of light stimulus
GO:0070838	115	56	1.12E-03	divalent metal ion transport
GO:0006940	16	12	1.12E-03	regulation of smooth muscle contraction
GO:0007409	305	131	1.15E-03	axonogenesis
GO:0048858	373	157	1.15E-03	cell projection morphogenesis
GO:0002695	30	19	1.17E-03	negative regulation of leukocyte activation
GO:0019722	30	19	1.17E-03	calcium-mediated signaling
GO:0009410	70	37	1.18E-03	response to xenobiotic stimulus
GO:0043269	77	40	1.19E-03	regulation of ion transport
GO:0009583	18	13	1.22E-03	detection of light stimulus
GO:0050728	18	13	1.22E-03	negative regulation of inflammatory response
GO:0050886	18	13	1.22E-03	endocrine process
GO:0048598	218	97	1.27E-03	embryonic morphogenesis
GO:0007193	24	16	1.30E-03	inhibition of adenylate cyclase activity by G-protein signaling pathway
GO:0009887	345	146	1.30E-03	organ morphogenesis
GO:0042445	82	42	1.30E-03	hormone metabolic process
GO:0003014	22	15	1.31E-03	renal system process
GO:0030193	22	15	1.31E-03	regulation of blood coagulation
GO:0003015	52	29	1.32E-03	heart process
GO:0060047	52	29	1.32E-03	heart contraction
GO:0006805	68	36	1.32E-03	xenobiotic metabolic process
GO:0032943	68	36	1.32E-03	mononuclear cell proliferation
GO:0046651	68	36	1.32E-03	lymphocyte proliferation
GO:0071466	68	36	1.32E-03	cellular response to xenobiotic stimulus
GO:0006873	244	107	1.36E-03	cellular ion homeostasis
GO:0050795	41	24	1.37E-03	regulation of behavior
GO:0072511	116	56	1.44E-03	divalent inorganic cation transport
GO:0048562	80	41	1.46E-03	embryonic organ morphogenesis
GO:0007565	66	35	1.47E-03	female pregnancy
GO:0007610	186	84	1.54E-03	behavior
GO:0010876	136	64	1.57E-03	lipid localization
GO:0009953	37	22	1.63E-03	dorsal/ventral pattern formation
GO:0022008	557	225	1.66E-03	neurogenesis
GO:0021892	6	6	1.68E-03	cerebral cortex GABAergic interneuron differentiation
GO:0034501	6	6	1.68E-03	protein localization to kinetochore
GO:0006869	124	59	1.69E-03	lipid transport
GO:0002376	774	305	1.72E-03	immune system process
GO:0045137	107	52	1.74E-03	development of primary sexual characteristics
GO:0019953	287	123	1.76E-03	sexual reproduction
GO:0046068	11	9	1.81E-03	cGMP metabolic process
GO:0051899	33	20	1.91E-03	membrane depolarization
GO:0007420	228	100	1.91E-03	brain development
GO:0050794	4968	1783	1.93E-03	regulation of cellular process
GO:0032990	377	157	1.96E-03	cell part morphogenesis
GO:0031175	385	160	1.98E-03	neuron projection development
GO:0021772	13	10	2.21E-03	olfactory bulb development

GO:0030728	13	10	2.21E-03	ovulation
GO:0045453	13	10	2.21E-03	bone resorption
GO:0030900	113	54	2.31E-03	forebrain development
GO:0007224	27	17	2.33E-03	smoothened signaling pathway
GO:0071702	323	136	2.35E-03	organic substance transport
GO:0048568	123	58	2.39E-03	embryonic organ development
GO:0001654	101	49	2.44E-03	eye development
GO:0050789	5203	1862	2.52E-03	regulation of biological process
GO:0007605	56	30	2.55E-03	sensory perception of sound
GO:0006775	23	15	2.56E-03	fat-soluble vitamin metabolic process
GO:0048489	23	15	2.56E-03	synaptic vesicle transport
GO:0007200	17	12	2.61E-03	activation of phospholipase C activity by G-protein coupled receptor protein signaling pathway coupled to IP3 second messenger
GO:0001894	38	22	2.62E-03	tissue homeostasis
GO:0046888	21	14	2.63E-03	negative regulation of hormone secretion
GO:0055082	251	108	2.78E-03	cellular chemical homeostasis
GO:0051252	1474	556	2.78E-03	regulation of RNA metabolic process
GO:0008406	97	47	3.05E-03	gonad development
GO:0034097	200	88	3.09E-03	response to cytokine stimulus
GO:0007018	102	49	3.14E-03	microtubule-based movement
GO:0048771	34	20	3.14E-03	tissue remodeling
GO:0030030	485	196	3.15E-03	cell projection organization
GO:0002504	8	7	3.24E-03	antigen processing and presentation of peptide or polysaccharide antigen via MHC class II
GO:0006182	8	7	3.24E-03	cGMP biosynthetic process
GO:0009584	8	7	3.24E-03	detection of visible light
GO:0043266	8	7	3.24E-03	regulation of potassium ion transport
GO:0045987	8	7	3.24E-03	positive regulation of smooth muscle contraction
GO:0048488	8	7	3.24E-03	synaptic vesicle endocytosis
GO:0046649	208	91	3.25E-03	lymphocyte activation
GO:0015849	112	53	3.28E-03	organic acid transport
GO:0001501	175	78	3.42E-03	skeletal system development
GO:0030510	32	19	3.43E-03	regulation of BMP signaling pathway
GO:0060429	250	107	3.55E-03	epithelium development
GO:0043583	64	33	3.60E-03	ear development
GO:0015908	30	18	3.73E-03	fatty acid transport
GO:0043010	76	38	3.74E-03	camera-type eye development
GO:0032879	573	228	3.75E-03	regulation of localization
GO:0046660	62	32	4.04E-03	female sex differentiation
GO:0031348	28	17	4.04E-03	negative regulation of defense response
GO:0051250	28	17	4.04E-03	negative regulation of lymphocyte activation
GO:0008585	55	29	4.07E-03	female gonad development
GO:0007548	123	57	4.18E-03	sex differentiation
GO:0019216	91	44	4.28E-03	regulation of lipid metabolic process
GO:0018298	10	8	4.34E-03	protein-chromophore linkage
GO:0045933	10	8	4.34E-03	positive regulation of muscle contraction
GO:0009880	26	16	4.36E-03	embryonic pattern specification
GO:0031644	67	34	4.39E-03	regulation of neurological system process
GO:0045321	241	103	4.40E-03	leukocyte activation
GO:0050804	60	31	4.53E-03	regulation of synaptic transmission
GO:0046942	111	52	4.61E-03	carboxylic acid transport
GO:0007631	24	15	4.66E-03	feeding behavior
GO:0031281	24	15	4.66E-03	positive regulation of cyclase activity
GO:0048583	1148	436	4.84E-03	regulation of response to stimulus
GO:2000026	446	180	4.85E-03	regulation of multicellular organismal development
GO:0014061	5	5	4.88E-03	regulation of norepinephrine secretion
GO:0014821	5	5	4.88E-03	phasic smooth muscle contraction
GO:0015874	5	5	4.88E-03	norepinephrine transport

GO:0021826	5	5	4.88E-03	substrate-independent telencephalic tangential migration
GO:0021843	5	5	4.88E-03	substrate-independent telencephalic tangential interneuron migration
GO:0030147	5	5	4.88E-03	natriuresis
GO:0035095	5	5	4.88E-03	behavioral response to nicotine
GO:0048243	5	5	4.88E-03	norepinephrine secretion
GO:0051552	5	5	4.88E-03	flavone metabolic process
GO:0007190	22	14	4.95E-03	activation of adenylate cyclase activity
GO:0018149	12	9	5.01E-03	peptide cross-linking
GO:0043113	12	9	5.01E-03	receptor clustering
GO:0048513	1168	443	5.02E-03	organ development
GO:0046545	58	30	5.08E-03	development of primary female sexual characteristics
GO:0003002	129	59	5.15E-03	regionalization
GO:0046883	82	40	5.18E-03	regulation of hormone secretion
GO:0001659	14	10	5.34E-03	temperature homeostasis
GO:0001893	14	10	5.34E-03	maternal placenta development
GO:0007602	14	10	5.34E-03	phototransduction
GO:0016079	14	10	5.34E-03	synaptic vesicle exocytosis
GO:0021988	14	10	5.34E-03	olfactory lobe development
GO:0048483	14	10	5.34E-03	autonomic nervous system development
GO:0015844	18	12	5.37E-03	monoamine transport
GO:0030195	18	12	5.37E-03	negative regulation of blood coagulation
GO:0007189	16	11	5.44E-03	activation of adenylate cyclase activity by G-protein signaling pathway
GO:0010578	16	11	5.44E-03	regulation of adenylate cyclase activity involved in G-protein signaling pathway
GO:0010579	16	11	5.44E-03	positive regulation of adenylate cyclase activity by G-protein signaling pathway
GO:0016525	16	11	5.44E-03	negative regulation of angiogenesis
GO:0035136	16	11	5.44E-03	forelimb morphogenesis
GO:0051952	16	11	5.44E-03	regulation of amine transport
GO:0009581	33	19	5.50E-03	detection of external stimulus
GO:0042391	92	44	5.50E-03	regulation of membrane potential
GO:0007156	102	48	5.69E-03	homophilic cell adhesion
GO:0072358	338	139	5.86E-03	cardiovascular system development
GO:0072359	338	139	5.86E-03	circulatory system development
GO:0001763	85	41	6.00E-03	morphogenesis of a branching structure
GO:0050878	357	146	6.11E-03	regulation of body fluid levels
GO:0002443	73	36	6.24E-03	leukocyte mediated immunity
GO:0031670	29	17	6.66E-03	cellular response to nutrient
GO:0061138	71	35	7.01E-03	morphogenesis of a branching epithelium
GO:0042060	377	153	7.09E-03	wound healing
GO:0015837	98	46	7.10E-03	amine transport
GO:0021953	45	24	7.16E-03	central nervous system neuron differentiation
GO:0070663	52	27	7.17E-03	regulation of leukocyte proliferation
GO:0042592	572	225	7.34E-03	homeostatic process
GO:0051969	64	32	7.46E-03	regulation of transmission of nerve impulse
GO:0030855	126	57	7.79E-03	epithelial cell differentiation
GO:0050818	25	15	7.97E-03	regulation of coagulation
GO:0051349	25	15	7.97E-03	positive regulation of lyase activity
GO:0032103	43	23	8.02E-03	positive regulation of response to external stimulus
GO:0032944	50	26	8.04E-03	regulation of mononuclear cell proliferation
GO:0050670	50	26	8.04E-03	regulation of lymphocyte proliferation
GO:0061061	206	88	8.15E-03	muscle structure development
GO:0007622	7	6	8.30E-03	rhythmic behavior
GO:0010677	7	6	8.30E-03	negative regulation of cellular carbohydrate metabolic process
GO:0019317	7	6	8.30E-03	fucose catabolic process
GO:0032715	7	6	8.30E-03	negative regulation of interleukin-6 production

GO:0042355	7	6	8.30E-03	L-fucose catabolic process
GO:0043383	7	6	8.30E-03	negative T cell selection
GO:0045005	7	6	8.30E-03	maintenance of fidelity involved in DNA-dependent DNA replication
GO:0045912	7	6	8.30E-03	negative regulation of carbohydrate metabolic process
GO:0048484	7	6	8.30E-03	enteric nervous system development
GO:0051279	7	6	8.30E-03	regulation of release of sequestered calcium ion into cytosol
GO:2000273	7	6	8.30E-03	positive regulation of receptor activity
GO:0040012	175	76	8.35E-03	regulation of locomotion
GO:0050708	34	19	8.48E-03	regulation of protein secretion
GO:0061180	34	19	8.48E-03	mammary gland epithelium development
GO:0007517	152	67	8.59E-03	muscle organ development
GO:0021675	23	14	8.66E-03	nerve development
GO:0045762	23	14	8.66E-03	positive regulation of adenylate cyclase activity
GO:0002790	89	42	8.77E-03	peptide secretion
GO:0002460	55	28	8.79E-03	adaptive immune response based on somatic recombination of immune receptors built from immunoglobulin superfamily domains
GO:0055065	142	63	8.97E-03	metal ion homeostasis
GO:0006875	137	61	9.15E-03	cellular metal ion homeostasis
GO:0021510	21	13	9.36E-03	spinal cord development
GO:0009582	32	18	9.43E-03	detection of abiotic stimulus
GO:0030048	32	18	9.43E-03	actin filament-based movement
GO:0050864	32	18	9.43E-03	regulation of B cell activation
GO:0048511	97	45	9.93E-03	rhythmic process

IE.3) Inconsistently Expressed Genes: Molecular Function

GOID	GO #gene	CE #gene	p-value	GOterm
GO:0004888	387	256	6.48E-38	transmembrane receptor activity
GO:0004872	607	357	4.09E-36	receptor activity
GO:0004930	169	128	4.20E-28	G-protein coupled receptor activity
GO:0004871	876	454	7.03E-28	signal transducer activity
GO:0060089	876	454	7.03E-28	molecular transducer activity
GO:0015267	191	123	2.67E-17	channel activity
GO:0022803	191	123	2.67E-17	passive transmembrane transporter activity
GO:0022838	186	120	5.31E-17	substrate-specific channel activity
GO:0005216	183	118	1.03E-16	ion channel activity
GO:0004984	28	28	1.18E-13	olfactory receptor activity
GO:0005261	127	84	3.43E-13	cation channel activity
GO:0022836	134	84	2.54E-11	gated channel activity
GO:0042165	32	28	8.47E-10	neurotransmitter binding
GO:0005179	31	27	2.15E-09	hormone activity
GO:0001653	36	30	2.41E-09	peptide receptor activity
GO:0008528	36	30	2.41E-09	peptide receptor activity, G-protein coupled
GO:0015276	50	38	2.53E-09	ligand-gated ion channel activity
GO:0022834	50	38	2.53E-09	ligand-gated channel activity
GO:0030594	29	25	1.38E-08	neurotransmitter receptor activity
GO:0022857	525	237	2.07E-07	transmembrane transporter activity
GO:0005230	23	20	3.19E-07	extracellular ligand-gated ion channel activity
GO:0005231	17	16	4.80E-07	excitatory extracellular ligand-gated ion channel activity
GO:0015075	405	187	6.57E-07	ion transmembrane transporter activity
GO:0022891	490	219	1.49E-06	substrate-specific transmembrane transporter activity
GO:0022843	63	40	2.64E-06	voltage-gated cation channel activity
GO:0004497	36	26	4.56E-06	monooxygenase activity

GO:0005262	46	31	5.77E-06	calcium channel activity
GO:0042923	17	15	7.53E-06	neuropeptide binding
GO:0008227	11	11	8.50E-06	G-protein coupled amine receptor activity
GO:0003774	114	62	1.09E-05	motor activity
GO:0005215	696	293	1.37E-05	transporter activity
GO:0004222	64	39	1.47E-05	metalloendopeptidase activity
GO:0005244	88	50	1.58E-05	voltage-gated ion channel activity
GO:0022832	88	50	1.58E-05	voltage-gated channel activity
GO:0008188	16	14	1.93E-05	neuropeptide receptor activity
GO:0046872	2761	1045	1.94E-05	metal ion binding
GO:0015293	71	42	1.97E-05	symporter activity
GO:0003777	65	39	2.46E-05	microtubule motor activity
GO:0043169	2788	1053	2.68E-05	cation binding
GO:0043167	2795	1055	2.95E-05	ion binding
GO:0019955	57	35	3.19E-05	cytokine binding
GO:0046914	1650	643	3.33E-05	transition metal ion binding
GO:0008270	1483	581	4.41E-05	zinc ion binding
GO:0022892	597	251	6.38E-05	substrate-specific transporter activity
GO:0001637	9	9	7.11E-05	G-protein chemoattractant receptor activity
GO:0004950	9	9	7.11E-05	chemokine receptor activity
GO:0015291	113	59	8.58E-05	secondary active transmembrane transporter activity
GO:0005267	55	33	1.02E-04	potassium channel activity
GO:0001871	77	43	1.06E-04	pattern binding
GO:0030247	77	43	1.06E-04	polysaccharide binding
GO:0008324	313	140	1.11E-04	cation transmembrane transporter activity
GO:0003677	1667	643	1.33E-04	DNA binding
GO:0005125	60	35	1.42E-04	cytokine activity
GO:0008081	56	33	1.67E-04	phosphoric diester hydrolase activity
GO:0005201	35	23	1.70E-04	extracellular matrix structural constituent
GO:0004435	16	13	1.79E-04	phosphatidylinositol phospholipase C activity
GO:0008237	113	58	1.80E-04	metallopeptidase activity
GO:0019956	11	10	1.85E-04	chemokine binding
GO:0030246	186	88	2.14E-04	carbohydrate binding
GO:0004629	20	15	2.68E-04	phospholipase C activity
GO:0004867	36	23	3.17E-04	serine-type endopeptidase inhibitor activity
GO:0001664	67	37	4.23E-04	G-protein-coupled receptor binding
GO:0016709	15	12	4.24E-04	oxidoreductase activity, acting on paired donors, with incorporation or reduction of molecular oxygen, NADH or NADPH as one donor, and incorporation of one atom of oxygen
GO:0005509	402	171	4.78E-04	calcium ion binding
GO:0016712	10	9	4.90E-04	oxidoreductase activity, acting on paired donors, with incorporation or reduction of molecular oxygen, reduced flavin or flavoprotein as one donor, and incorporation of one atom of oxygen
GO:0042166	10	9	4.90E-04	acetylcholine binding
GO:0005539	72	39	5.05E-04	glycosaminoglycan binding
GO:0004889	7	7	5.95E-04	nicotinic acetylcholine-activated cation-selective channel activity
GO:0008417	12	10	7.64E-04	fucosyltransferase activity
GO:0016298	53	30	8.40E-04	lipase activity
GO:0005102	533	219	8.49E-04	receptor binding
GO:0005342	60	33	9.37E-04	organic acid transmembrane transporter activity
GO:0005272	16	12	1.16E-03	sodium channel activity
GO:0004620	45	26	1.22E-03	phospholipase activity
GO:0019840	18	13	1.27E-03	isoprenoid binding
GO:0008066	9	8	1.28E-03	glutamate receptor activity
GO:0015464	9	8	1.28E-03	acetylcholine receptor activity
GO:0005249	43	25	1.33E-03	voltage-gated potassium channel activity
GO:0030695	355	150	1.44E-03	GTPase regulator activity
GO:0046943	59	32	1.52E-03	carboxylic acid transmembrane transporter activity

GO:0004970	6	6	1.72E-03	ionotropic glutamate receptor activity
GO:0005234	6	6	1.72E-03	extracellular-glutamate-gated ion channel activity
GO:0016493	6	6	1.72E-03	C-C chemokine receptor activity
GO:0019957	6	6	1.72E-03	C-C chemokine binding
GO:0005083	237	104	1.76E-03	small GTPase regulator activity
GO:0005085	119	57	1.82E-03	guanyl-nucleotide exchange factor activity
GO:0005516	100	49	2.05E-03	calmodulin binding
GO:0005501	17	12	2.70E-03	retinoid binding
GO:0060589	365	152	2.71E-03	nucleoside-triphosphatase regulator activity
GO:0004714	47	26	2.85E-03	transmembrane receptor protein tyrosine kinase activity
GO:0008138	36	21	3.02E-03	protein tyrosine/serine/threonine phosphatase activity
GO:0061134	87	43	3.04E-03	peptidase regulator activity
GO:0070330	8	7	3.32E-03	aromatase activity
GO:0022804	221	96	3.74E-03	active transmembrane transporter activity
GO:0046920	5	5	4.97E-03	alpha(1,3)-fucosyltransferase activity
GO:0071855	5	5	4.97E-03	neuropeptide receptor binding
GO:0005044	22	14	5.14E-03	scavenger receptor activity
GO:0008028	12	9	5.15E-03	monocarboxylic acid transmembrane transporter activity
GO:0008373	12	9	5.15E-03	sialyltransferase activity
GO:0019825	12	9	5.15E-03	oxygen binding
GO:0030414	65	33	5.25E-03	peptidase inhibitor activity
GO:0015020	18	12	5.55E-03	glucuronosyltransferase activity
GO:0001071	624	246	5.63E-03	nucleic acid binding transcription factor activity
GO:0005089	56	29	6.03E-03	Rho guanyl-nucleotide exchange factor activity
GO:0042578	258	109	6.05E-03	phosphoric ester hydrolase activity
GO:0005088	68	34	6.31E-03	Ras guanyl-nucleotide exchange factor activity
GO:0046906	68	34	6.31E-03	tetrapyrrole binding
GO:0003700	623	245	6.56E-03	sequence-specific DNA binding transcription factor activity
GO:0005275	47	25	6.74E-03	amine transmembrane transporter activity
GO:0015081	45	24	7.53E-03	sodium ion transmembrane transporter activity
GO:0015171	43	23	8.42E-03	amino acid transmembrane transporter activity
GO:0015245	7	6	8.47E-03	fatty acid transporter activity
GO:0015370	23	14	8.98E-03	solute
