# Supplementary material for: Single cell transcriptome analysis of MCF-7 reveals consistently and inconsistently expressed gene groups each associated with distinct cellular localization and functions
Source: PLoS One. 2018 Jun 19;13(6):e0199471. doi: 10.1371/journal.pone.0199471 (PMC6007910; doi:10.1371/journal.pone.0199471)
Supplement: S3 Table — (Pathway analysis for IE is skipped because they are mainly extracellular proteins). (PDF) [file pone.0199471.s003.pdf]

Supplementary Table S3:
Pathway analysis of CE genes using BioCarta pathway database

Pathway database: BioCarta				
Total number gene in BioCarta: 29,200				
Total number of CE genes: 8,653				
P-value cutoff: 0.001				
**# pathway genes**	**# annotated genes**	**%**	**p-value**	**pathway name**
23	23	100%	0	MTOR_PATHWAY
87	66	76%	0	MAPK_PATHWAY
19	19	100%	0	PROTEASOME_PATHWAY
11	11	100%	0	EIF2_PATHWAY
8	8	100%	0	KREB_PATHWAY
40	32	80%	6.23E-12	P38MAPK_PATHWAY
24	22	92%	1.20E-11	EIF4_PATHWAY
37	30	81%	1.25E-11	MET_PATHWAY
20	19	95%	2.69E-11	IGF1MTOR_PATHWAY
32	26	81%	2.00E-10	PDGF_PATHWAY
18	17	94%	3.07E-10	PTEN_PATHWAY
29	24	83%	3.79E-10	PYK2_PATHWAY
31	25	81%	5.73E-10	EGF_PATHWAY
26	22	85%	6.65E-10	RACCYCD_PATHWAY
26	22	85%	6.65E-10	WNT_PATHWAY
23	20	87%	1.04E-09	GLEEVEC_PATHWAY
23	20	87%	1.04E-09	IGF1R_PATHWAY
20	18	90%	1.30E-09	ACTINY_PATHWAY
22	19	86%	3.20E-09	CERAMIDE_PATHWAY
22	19	86%	3.20E-09	IL6_PATHWAY
16	15	94%	3.50E-09	EIF_PATHWAY
16	15	94%	3.50E-09	CDC42RAC_PATHWAY
38	28	74%	3.69E-09	INTEGRIN_PATHWAY
29	23	79%	4.61E-09	VEGF_PATHWAY
21	18	86%	9.85E-09	IGF1_PATHWAY
38	27	71%	2.60E-08	IL2RB_PATHWAY
44	30	68%	2.66E-08	CHREBP2_PATHWAY
22	18	82%	5.20E-08	INSULIN_PATHWAY

24	19	79%	7.61E-08	TPO_PATHWAY
28	21	75%	1.21E-07	ERK_PATHWAY
34	24	71%	1.58E-07	AT1R_PATHWAY
23	18	78%	2.15E-07	RAS_PATHWAY
18	15	83%	2.79E-07	ERK5_PATHWAY
58	35	60%	3.06E-07	PPARA_PATHWAY
27	20	74%	3.23E-07	GSK3_PATHWAY
27	20	74%	3.23E-07	CREB_PATHWAY
39	26	67%	3.76E-07	FCER1_PATHWAY
35	24	69%	3.98E-07	BCR_PATHWAY
35	24	69%	3.98E-07	GPCR_PATHWAY
12	11	92%	4.56E-07	ETC_PATHWAY
24	18	75%	7.44E-07	CXCR4_PATHWAY
17	14	82%	8.40E-07	HCMV_PATHWAY
28	20	71%	9.28E-07	GH_PATHWAY
34	23	68%	9.67E-07	MPR_PATHWAY
58	34	59%	1.17E-06	HIVNEF_PATHWAY
19	15	79%	1.28E-06	EPO_PATHWAY
19	15	79%	1.28E-06	MAL_PATHWAY
43	27	63%	1.57E-06	BIOPEPTIDES_PATHWAY
37	24	65%	2.07E-06	FMLP_PATHWAY
35	23	66%	2.21E-06	CARM_ER_PATHWAY
16	13	81%	2.51E-06	BCELLSURVIVAL_PATHWAY
16	13	81%	2.51E-06	CDMAC_PATHWAY
18	14	78%	3.65E-06	NGF_PATHWAY
13	11	85%	4.31E-06	P27_PATHWAY
20	15	75%	4.61E-06	ATM_PATHWAY
47	28	60%	5.11E-06	TCR_PATHWAY
10	9	90%	5.20E-06	RANMS_PATHWAY
22	16	73%	5.32E-06	HER2_PATHWAY
24	17	71%	5.77E-06	G2_PATHWAY
24	17	71%	5.77E-06	ECM_PATHWAY
28	19	68%	5.97E-06	G1_PATHWAY
15	12	80%	7.46E-06	HIF_PATHWAY
15	12	80%	7.46E-06	PITX2_PATHWAY
15	12	80%	7.46E-06	RARRXR_PATHWAY
19	14	74%	1.25E-05	MTA3_PATHWAY

19	14	74%	1.25E-05	TGFB_PATHWAY
12	10	83%	1.35E-05	VDR_PATHWAY
12	10	83%	1.35E-05	RAB_PATHWAY
21	15	71%	1.40E-05	TFF_PATHWAY
23	16	70%	1.47E-05	CTCF_PATHWAY
14	11	79%	2.19E-05	PS1_PATHWAY
32	20	62%	2.71E-05	RHO_PATHWAY
30	19	63%	3.00E-05	HDAC_PATHWAY
18	13	72%	3.37E-05	ARAP_PATHWAY
18	13	72%	3.37E-05	TEL_PATHWAY
22	15	68%	3.71E-05	CHEMICAL_PATHWAY
46	26	57%	3.90E-05	KERATINOCYTE_PATHWAY
11	9	82%	4.18E-05	P35ALZHEIMERS_PATHWAY
13	10	77%	6.39E-05	CARM1_PATHWAY
29	18	62%	6.83E-05	TNFR1_PATHWAY
15	11	73%	7.99E-05	IL3_PATHWAY
15	11	73%	7.99E-05	AKAPCENTROSOME_PATHWAY
25	16	64%	8.25E-05	STRESS_PATHWAY
23	15	65%	8.83E-05	P53HYPOXIA_PATHWAY
23	15	65%	8.83E-05	PTDINS_PATHWAY
17	12	71%	8.92E-05	ARF_PATHWAY
21	14	67%	9.22E-05	MEF2D_PATHWAY
21	14	67%	9.22E-05	ATRBRCA_PATHWAY
10	8	80%	1.29E-04	RNA_PATHWAY
10	8	80%	1.29E-04	GLYCOLYSIS_PATHWAY
37	21	57%	1.55E-04	PAR1_PATHWAY
26	16	62%	1.73E-04	BAD_PATHWAY
12	9	75%	1.84E-04	BARR_MAPK_PATHWAY
12	9	75%	1.84E-04	TRKA_PATHWAY
24	15	62%	1.92E-04	NTHI_PATHWAY
22	14	64%	2.10E-04	IL2_PATHWAY
16	11	69%	2.33E-04	RELA_PATHWAY
18	12	67%	2.34E-04	ETS_PATHWAY
27	16	59%	3.37E-04	VIP_PATHWAY
27	16	59%	3.37E-04	EDG1_PATHWAY
21	13	62%	4.90E-04	MITOCHONDRIA_PATHWAY
54	27	50%	5.08E-04	NFAT_PATHWAY

11	8	73%	5.21E-04	EPONFKB_PATHWAY
11	8	73%	5.21E-04	CDK5_PATHWAY
30	17	57%	5.28E-04	FAS_PATHWAY
19	12	63%	5.39E-04	CALCINEURIN_PATHWAY
13	9	69%	5.83E-04	ARENRF2_PATHWAY
13	9	69%	5.83E-04	RB_PATHWAY
15	10	67%	5.96E-04	BARRESTIN_SRC_PATHWAY
15	10	67%	5.96E-04	LONGEVITY_PATHWAY
31	17	55%	9.11E-04	MYOSIN_PATHWAY
22	13	59%	9.81E-04	AKT_PATHWAY
20	12	60%	1.12E-03	GCR_PATHWAY
20	12	60%	1.12E-03	NKCELLS_PATHWAY
18	11	61%	1.26E-03	TNFR2_PATHWAY
18	11	61%	1.26E-03	UCALPAIN_PATHWAY
16	10	62%	1.40E-03	P53_PATHWAY
10	7	70%	1.45E-03	DNAFRAGMENT_PATHWAY
10	7	70%	1.45E-03	SKP2E2F_PATHWAY
